# Metabolite profiling of *Dioscorea* (yam) species reveals underutilised biodiversity and renewable sources for high-value compounds

**DOI:** 10.1038/srep29136

**Published:** 2016-07-07

**Authors:** Elliott J. Price, Paul Wilkin, Viswambharan Sarasan, Paul D. Fraser

**Affiliations:** 1 School of Biological Sciences, Royal Holloway University of London, Egham, Surrey, TW20 0EX, UK; 2 Royal Botanic Gardens, Kew, Richmond, Surrey, TW20 3AB, UK

## Abstract

Yams (*Dioscorea spp.*) are a multispecies crop with production in over 50 countries generating ~50 MT of edible tubers annually. The long-term storage potential of these tubers is vital for food security in developing countries. Furthermore, many species are important sources of pharmaceutical precursors. Despite these attributes as staple food crops and sources of high-value chemicals, *Dioscorea spp.* remain largely neglected in comparison to other staple tuber crops of tropical agricultural systems such as cassava (*Manihot esculenta*) and sweet potato (*Ipomoea batatas*). To date, studies have focussed on the tubers or rhizomes of *Dioscorea*, neglecting the foliage as waste. In the present study metabolite profiling procedures, using GC-MS approaches, have been established to assess biochemical diversity across species. The robustness of the procedures was shown using material from the phylogenetic clades. The resultant data allowed separation of the genotypes into clades, species and morphological traits with a putative geographical origin. Additionally, we show the potential of foliage material as a renewable source of high-value compounds.

*Dioscorea* (yam) species comprise a genus of 613 tuberous climbing plants^1^. Of these, seven to ten are cultivated on a large scale^2^,^3^ and two (*D. alata*, *D. cayennensis* Lam. subsp. *cayennensis* and *D. cayenennsis Lam.* subsp. *rotundata* (Poir.) J. Miège [referred to *D. rotundata* throughout]) are of primary importance as a staple crop, predominately in Western Africa^4^, for over 100 million people^5^. Approximately a further 50 species are eaten as wild-harvested staples or famine food and the genus holds importance for global food security^6^. In addition, *Dioscorea* species have been widely used in traditional medicines^7^,^8^ and as a source of steroidal precurors^9^,^10^. Nevertheless yams are categorised as understudied and underutilised^11^,^12^.

Yam production is relatively expensive compared to other root and tuber crops due to high planting and labour costs, a long growing season and low yield per hectare^3^,^13^. The cost per 1000 calories from yam is estimated at 4 times that for cassava (*Manihot esculenta Crantz*)^14^. Yams hold cultural and social importance^11^ and have preferred organoleptic properties compared with other carbohydrate sources, including cassava, potatoes (within *Solanum spp.*) and sweet potato (*Ipomoea batatas* (L.) Lam.)^15^. Sensorial preference, coupled with better storage qualities compared to crops such as cassava and plantain (*Musa spp.*)^16^, have led to high-demand as a cash crop^13^.

The abundance of steroidal C_27_ saponins in the rhizome or tubers of some *Dioscorea* has long been known^8^,^17^,^18^,^19^. Diosgenin, the aglycone portion of the abundant saponin dioscin, has been industrially exploited as the starting material for the synthesis of pregnenolone-derived steroids^10^. Around 15 species of *Dioscorea* are used as a source of diosgenin^20^; with an estimated market value of $500 million^21^. Over-harvesting of rare yams has threatened populations of several *Dioscorea* species worldwide^18^,^22^,^23^,^24^.

Use of *Dioscorea* species in traditional medicines is documented to at least 2000 BC^25^, including leaf material^26^ as a constituent. Species such as *D. dumetorum* and *D. hispida* Dennst. and their relatives have been those primarily been used for their poisonous properties, due to the presence of polar alkaloids^8^. Despite this, phytochemical analysis using metabolite profiling has not previously been conducted on the foliage of *Dioscorea* species in comparative biodiversity research.

The foliage material of yam is an agricultural waste product yet some species generate substantial above-ground biomass each annual growth cycle. Utilising *Dioscorea* foliage has potential to provide a renewable source of high-value natural products whilst sustaining conservation of species.

Lebot *et al*. applied a metabolite profiling approach to tubers of some tropical root crops including selected *Dioscorea* species, where results showed inter- and intra- species diversity in *Dioscorea*^27^,^28^, emphasising the potential of a more global metabolomic investigation.

In this study, we analysed the polar extracts from leaf and petiole material of a diverse collection of *Dioscorea* via Gas Chromatography-Mass Spectrometry (GC-MS). The metabolite profiles obtained enabled investigation of species discrimination and comparison with phylogenetic relationships and morphological characteristics. The profiles provide insight into biochemically-related species and highlight *Dioscorea* species as potential sources of valuable compounds.

## Results

### The yam metabolome

The devised platform for analysis of *Dioscorea* material (Supplementary Fig. S1) provided broad coverage of primary metabolism. Metabolic pathways which can be analysed by this platform include carbohydrate metabolism and amino acid biosynthesis, along with some nucleotides, secondary metabolites, monoamines and derivatives. A strong linear response (R^2^ > 0.98) was shown for 41 compounds measured (Supplementary Table S2).

Recovery rates for many compounds however, were low (<70% of the total extracted via three extractions, Supplementary Table S3). There were no qualitative changes (new metabolites extracted) following multiple extractions on samples, and thus one extraction is robust for fast screening. Overloading of the MS was necessary to achieve acceptable coverage of the metabolome and enhance the dynamic range.

A diagrammatic workflow of methods is provided in Supplementary Fig. S1.

### Species demarcation

**Figure 1:**
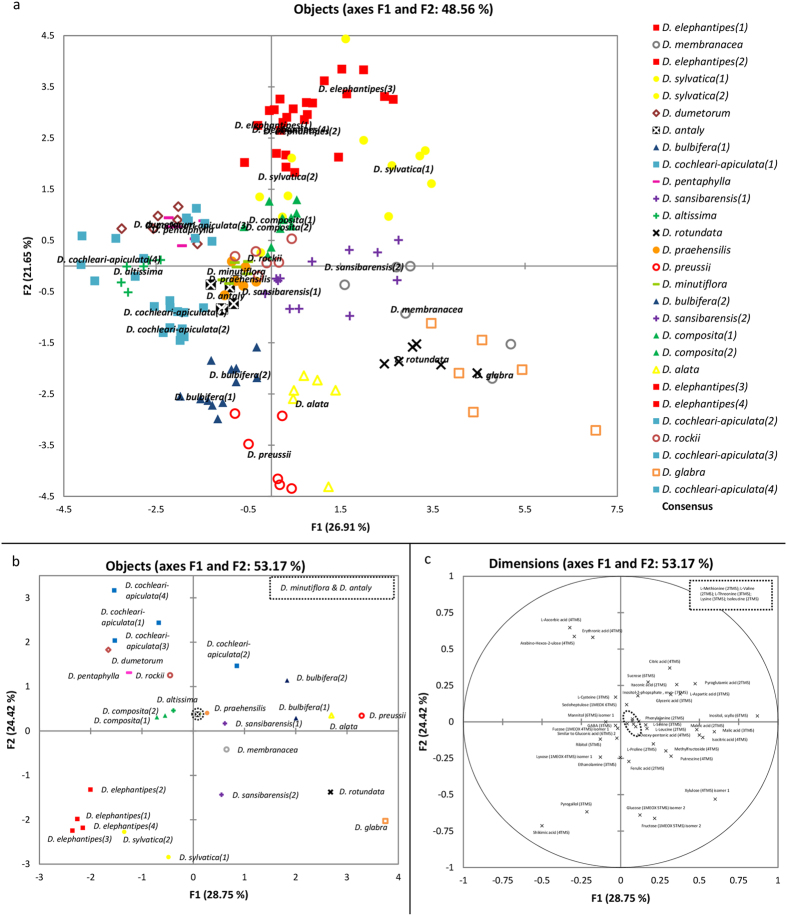
Generalised Procrustes Analysis on the polar fraction of metabolite extracts from leaf of *Dioscorea*, analysed by Gas Chromatography-Mass Spectrometry, provides (**a**) a transformed configuration from all six replicate analyses [Rc = 0.847 (100^th^ percentile); F1 = 78.998, F2 = 72.105 (p < 0.0001)] and (**b**) the consensus configuration from a reduced dataset of the 41 most discriminatory variables, with loadings shown in (**c**); which shows the same trends [Rc = 0.898 (100^th^ percentile); F1 = 85.499, F2 = 64.471 (p < 0.0001)].

Profiling of the polar extracts from foliage material of 28 *Dioscorea* accessions, covering 19 species (Table 1), allowed consistent measurement of 151 features (Supplementary Table S4). Clustering of replicates through GPA (Fig. 1a) highlighted the robustness of analysis and the consensus arrangement described 85% of total variation. Univariate analysis to identify the most discriminatory variables allowed reduction of the dataset to 41 variables, which enabled a comparable degree of species demarcation as using all 151 features (Fig. 1b, Supplementary Fig. S5).

Species tended to group on the basis of phylogenetic and/or morphological traits. Species of the African (Afr) clade (comprising *D. elephantipes* and *D. sylvatica*) formed a distinct group characterised by abundance of shikimic acid and pyrogallol. The majority of compound leaved (CL) species (*D. pentaphylla*, *D. cochleari-apiculata* & *D. dumetorum*) migrated towards sucrose, citric acid; ascorbic acid and its degradation product erythronic acid to form a cluster. Exceptions to this were *D. bulbifera* and *D. antaly*.

*D. bulbifera* showed a profile more similar to the cultivated species of Enantiophyllum e.g. *D. alata* and *D. rotundata* (in the same plane on F1, Fig. 1b & F2, Fig. 1a) whereas *D. antaly* clustered with crop wild relatives of *D. rotundata* (*D. praehensilis* and *D. minutiflora*) and Stenophora lineages (*D. membranacea*, *D*. *rockii*) around the origin of the GPA. Species from the New World (*D. composita* and *D. altissima*) also clustered around the origin of the plot in the reduced dataset (Fig. 1b), yet *D. altissima* clustered with CL species on the total GPA (Fig. 1a). Species at the origin all presented higher levels of amino acids and monosaccharides (Fig. 1c).

*D. alata and D. preussii* (both Enantiophyllum) migrate from the origin, primarily due to the influence of scyllo-inositol (Fig. 1b,c). Glucose, fructose and xylulose are the predominant variables distinguishing *D. rotundata* from its crop wild relatives: *D. praehensilis* & *D. minutiflora* (all three Enantiophyllum). Samples of *D. sansibarensis* (Malagasy) were distinguishable on F2, yet not F1 (Fig. 1b) with higher sugar content in one sample driving the separation.

**Figure 2:**
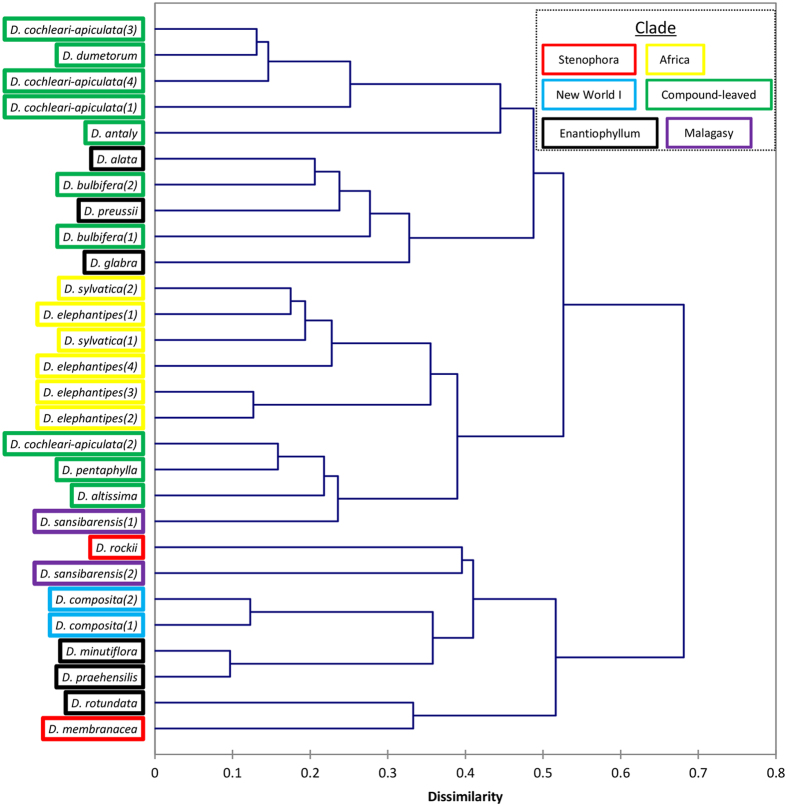
Hierarchical tree of *Dioscorea* accessions based on mean (n = 6) metabolite compositions shows relationship of chemotaxonomy with phylogenetic clades. Notably *D. rotundata* and crop-wild relatives (*D. praehensilis* and *D. minutiflora*) cluster with basal lineages of the Stenophora and New World I clades. Metabolite clustering is provided in a heat-map format in Supplementary Fig. S6.

Cluster analysis highlighted the affinity of *D. rotundata* and its crop wild relatives (*D. praehensilis* and *D. minutiflora*) with the most basal lineages of *Dioscorea* (Stenophora and New World clades) (Fig. 2). Additionally, the Afr clade formed a tight cluster flanked by groupings of CL species, with some outliers, e.g. the South American species *D. altissima.* Cluster analysis on the metabolites showed that biochemically-related compounds tended to group (Supplementary Fig. S6), supporting the reliability of the approach. *D. pentaphylla* could be distinguished from other CL species due to relatively high abundance of dopamine and the derivative norepinephrine and *D. antaly* was distinguishable due to relatively high levels of catechins (catechin, epicatechin and gallocatechin; Supplementary Fig. S6).

The demarcation of accessions is robust even with the limited tissue availability and cultivation conditions used.

### Compound atlas

**Figure 3:**
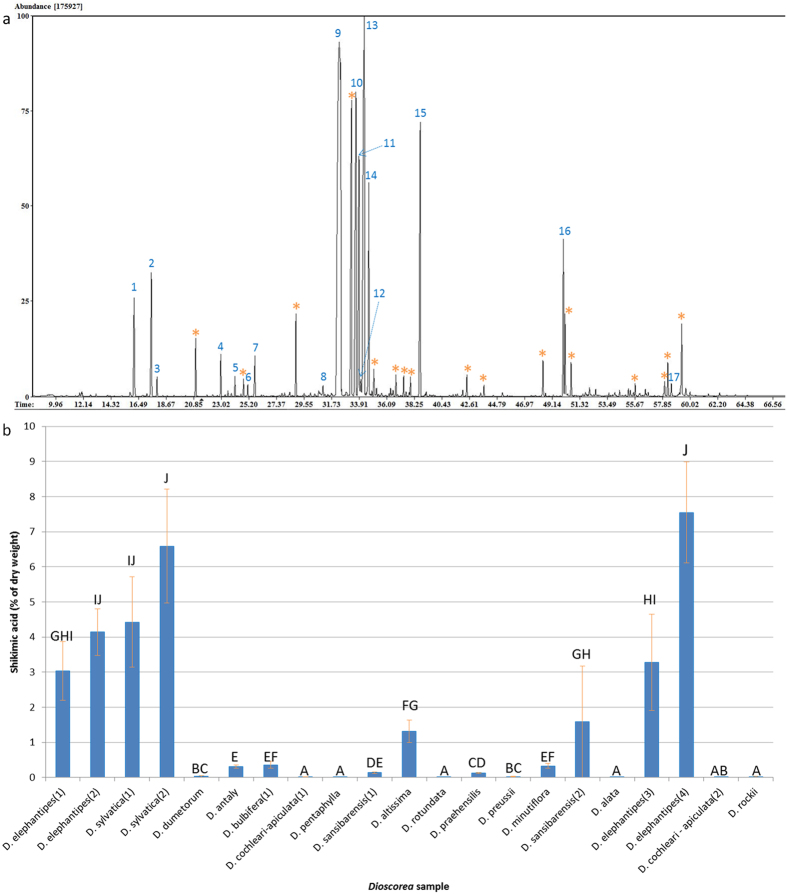
GC-MS analysis of *D. elephantipes* leaf material shows that (**a**) shikimic acid (4TMS) is often the most abundant peak recorded and (**b**) is significantly more abundant in species of the African clade. (**a**) Abundant peaks are: 1: Phosphate (3TMS), 2: Succinic-D_4_ acid (internal standard), 3: MSTFA, 4: Malic acid (3TMS), 5: GABA (3TMS), 6: Threonic acid (4TMS) 7: Xylulose (4TMS) isomer 1, 8: Methylfructofuranoside (4TMS), 9: Shikimic acid (4TMS), 10: Fructose (1MEOX 5TMS) isomer 1, 11: Fructose (1MEOX 5TMS) isomer 2, 12: Galactose (1MEOX 5TMS) isomer 1, 13: Glucose (1MEOX 5TMS) isomer 1, 14: Glucose (1MEOX 5TMS) isomer 2, 15: Inositol, myo (5TMS), 16: Sucrose (8TMS), 17: Melibiose (8TMS). Many major unknowns (*) are also present. (**b**) Groups from Bonferroni-corrected Conover-Iman post hoc following Kruskal-Wallis’. Error bars show 1 standard deviation (n = 6).

The polar extracts from leaf material of many *Dioscorea* species showed numerous unknown compounds and many of these were abundant. Using *D. elephantipes* as an example (Fig. 3a) a more detailed investigation of the metabolome was conducted. Both polar and non-polar phases were analysed and all features, including unknowns, were measured on different structures of the plant (stem, leaf, root, inner and outer of parts of caudiciform tuber). Comprehensive coverage (totalling 535 features) showed similar discrimination as when using the 206 known and putative metabolites and solely the 121 identified features (Supplementary Fig. S7). Further reduction of this dataset by choosing the most discriminatory variables (5 or more groups following Kruskal-Wallis’ and Conover-Iman post hoc) gave 38 features which can be used to discriminate regions of a single plant (Supplementary Fig. S7).

Shikimic acid was present in all regions in descending order: inner tuber, stem, leaf, outer tuber, roots. Sections were characterised by high abundance of particular metabolites. The inner caudex with amino acids, roots and outer caudex with trehalose and mannitol respectively and the leaf and stem with fructose, melibiose, GABA and erythronic acid.

### Shikimic acid quantification

To validate the high level of shikimic acid in foliage of *D. elephantipes* & *D. sylvatica*, further material was sourced, along with another caudiciform species: *D. mexicana.* Samples of *D. elephantipes* and *D. sylvatica* showed similarly abundant shikimic acid as previously, however only trace amounts were present in *D. mexicana*.

An authentic standard showed a good linear response (R^2^ = 0.9822, y = 0.1923x), relative to the internal standard, over the range 10–200 μg, with values in that of *D. elephantipes* and *D. sylvatica* approaching 8% (Fig. 3b).

## Discussion

Metabolite profiling via GC-MS is considered the gold standard technique for metabolite analysis^29^ providing good resolution, high reproducibility^30^ and can achieve broad compound coverage^31^. Equipment is relatively affordable and widely used ensuring a wealth of available resources such as established protocols^32^, large compound libraries^33^ and analysis software^31^. In comparison to other common analytical platforms GC-MS is more targeted towards intermediary metabolism with reliable compound identification. Therefore, a GC-MS based approach was favoured for initial biodiversity study of the *Dioscorea* metabolome.

A robust method (Supplementary Fig. S1) has been developed for metabolite analysis of *Dioscorea* species and applied to a diverse set of species and different plant organs. The approach provides representative coverage of the polar intermediary metabolome and can be easily extended to incorporate non-polar profiling. A low recovery rate was obtained for many compounds which may be due to the high starch (and sugar) content of material compromising extraction efficiency. Despite this, repeatable relative quantification can be achieved, evidenced by clustering of replicate samples analysed two months apart.

Sub-selections of metabolites can be identified which allows screening of small amounts of material (Fig. 1b, Supplementary Fig. S5) whilst remaining representative of total intermediary polar metabolism, as measured via this GC-MS methodology (Supplementary Fig. S7). Thus, a core set of small molecules could be defined to allow simplified rapid screening of *Dioscorea*.

The verification of *Dioscorea* species is often noted to be problematic^34^,^35^. Furthermore, a recent genotyping by sequencing (GBS) analysis could not discriminate Guinea yam species unless combined with ploidy analysis^36^. Metabolomics can aid both identification and also assess biochemical diversity concurrently, which is extremely beneficial to ongoing breeding programs. Data generated from the developed platform showed that related species cluster (e.g. *D. elephantipes* with *D. sylvatica*; *D. dumetorum* with *D. cochleari-apiculata*) and that the little studied species *D. altissima* clustered with *D. pentaphylla* and the other CL species (Fig. 2). Interestingly, this matches the topology of an unpublished phylogenetic tree based on plastid marker data (personal communication, P. Wilkin), supporting the idea that a metabolomics approach can be used in place of or alongside conventional morphological descriptors used for characterisation^37^.

**Figure 4:**
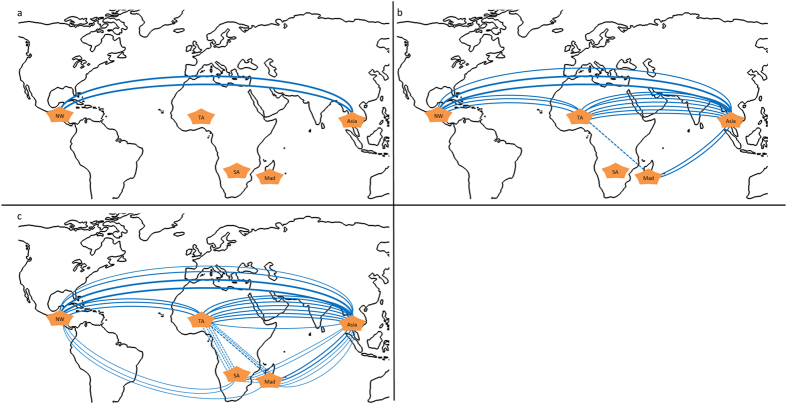
World map showing relationships between *Dioscorea* species from Asia (Asia) to the New World (NW), Tropical Africa (TA), South Africa (SA) and Madagascar (Mad) based on (**a**) first-degree, (**b**) second-degree and (**c**) third-degree linkages of samples following clustering on species-averaged metabolite compositions. Inter-continental transport is shown by dotted lines. World maps (from https://openclipart.org/) were modified in Microsoft Powerpoint.

Well-sampled phylogenetic study suggest that Stenophora and New World clades are the most basal^35^,^38^. Within this work, species of these clades appear to be a centre of biochemical origin (mostly central in the GPA, Fig. 1). Relationships of cluster analysis indicate, as previously hypothesised, that *Dioscorea* originated in Asia with early transfer to the New World^38^ (Fig. 4). Additionally, profiles obtained for CL and Afr clades are largely distinct. Species of the Enantiophyllum clades form a larger cluster overlapping other clades, which is not a surprise given that they are the youngest evolutionary lineages and inhabit a large geographical area.

Notably, *D. rotundata* and its crop wild relatives (*D. minutiflora* and *D. praehensilis*) have similar biochemical profiles to species of the Stenophora clade (Supplementary Fig. S6) and thus suggest the occurrence of convergent evolution. Around 90% of basal Stenophora species are distributed in Asia^39^, yet none in Africa where the central breeding programs of *Dioscorea* are based. Therefore, international co-operation will be greatly important for future breeding of these crops and capturing of traits from basal lineages.

A large proportion of unidentified metabolites have been measured in this study many of which are abundant and highlights the understudied nature of the genus. However, the genus may have potential for bioprospecting based on the finding of abundance of shikimic acid in stem and leaf material of *D. elephantipes* and *D. sylvatica* in this study.

Shikimic acid plays a central role in biosynthesis of aromatic amino acids in plants, fungi and bacteria and as such is measured in many metabolomics studies. However, high abundance of shikimic acid within the leaf material of *Dioscorea* accessions has not previously been reported. A precursor used in the production of the anti-viral oseltavimir (Tamiflu^®^), shikimic acid is typically sourced from the fruits of Chinese star anise (*Illicium verum*). Levels found in foliage of *D. elephantipes* and *D. sylvatica* are approaching those for Chinese star anise^40^ (Fig. 3b). Both species are under threat due to harvesting of tuber for traditional medicines^41^. The annual foliage is deemed waste material and can be used as an alternative source of shikimic acid which may promote the conservation and sustainable utilisation of these threatened wild species. The same could also be applied to other species of the genus offering a sustainable alternative to the destructive harvesting of tubers. Breeding for hybrids that produce better quality aerial biomass for less managed agroecosystems or as cover crops can improve livelihoods of farmers. Likewise selected clones can be mass propagated, using micropropagation, for industrial use and could potentially provide a profitable by-product for African yam production.

Initially, it was hypothesised that the abundance of shikimic acid may be related to tuber morphology yet only trace amounts were detected in *D. mexicana,* a caudiciform species of the New World. However, the hypothesis cannot be discarded as only one independent biological replicate could be sourced.

Detailed study on *D. elephantipes* showed that different plant organs are easily definable by very few highly accumulated metabolites. The roots and outer caudex were abundant with trehalose and mannitol, both of which have high water retention capacities and may contribute to the high drought tolerance of this species and GABA in above ground foliage may represent a response to such stress.

Additionally, *D. pentaphylla* was shown to contain abundant dopamine yet due to a lack of availability for further material these findings could not be verified. However, the species may be a potential source for catecholamine-derived pharmaceuticals and thus a good candidate for bioprospecting.

GPA has been shown to be an effective approach for analysis of metabolomics data, especially useful in studies when representative QC material is not available for scaling of data (Fig. 1), such as when sample material is limited or not available to be pooled prior to screening, often the case in large-scale studies. Also, when comparisons across sample sets are taken at different times, e.g. over multiple growth seasons, GPA removes the need for timely scaling of samples to QC’s. Additionally, different measurements, e.g. from improved compound libraries, can be integrated with previous data without the need to reanalyse all previous sample sets.

The platform represents progress for *Dioscorea* with potential to aid other studies, re-interpretation of historic data and implementation in breeding programs. Use of this GC-MS platform could be widely applied as cost is not prohibitive for developing countries growing yams (when compared to liquid chromatography (LC)-MS and other approaches). The ease of use and transferability of *Dioscorea*-specific compound libraries can provide the basis for metabolomics platform within breeding programs and allows the identification of diverse lineages.

Within this study robust results were shown from species grown under different glasshouse conditions. Future studies could use this metabolomics platform to investigate the influence of environmental factors on the metabolome and if the genetic determinates can overcome this influence.

Additionally, the many unknown abundant compounds in species (exampled Fig. 3a) highlight the further work required but provide potential leads for bioprospecting of this crop. The platform has been designed to allow extended analysis of non-polar^42^ and secondary metabolites on other platforms^43^ (e.g. LC -Photodiode Array (PDA)/-MS) from the same sample. This may prove useful, especially for species of the Stenophora and Compound-leaved clades which are widely utilised due to their high sterol and alkaloidal contents respectively. Therefore, the platform provides a basis for more holistic biochemical understanding of the economically, nutritionally and medicinally important yet understudied genus *Dioscorea*.

## Methods

### Reagents

All reagents were of analytical grade.

### Plant material

*Dioscorea* material was sourced from the Royal Botanic Gardens, Kew (Kew) Living Collections (http://epic.kew.org/index.htm) (Table 1). Youngest mature leaf and petiole material was sampled. Materials were cut from the vine and quenched in liquid nitrogen immediately before samples were lyophilised, homogenised and stored at −80 °C until further processing.

Additional dried leaf material of *D. elephantipes* (accessions 19900643A & 19280228C) and *D. sylvatica* (accession 19803437A) were sourced from the Royal Botanic Garden Edinburgh (RBGE) Living Collections (http://elmer.rbge.org.uk/bgbase/livcol/bgbaselivcol.php). Leaf material of *D. mexicana* Scheidw. (accession 8813370) was received from the Sukkulenten-Sammlung Zürich (https://www.stadt-zuerich.ch/sukkulenten).

For the compound atlas, a single commercially acquired *D. elephantipes* was used. The plant was halved and samples taken from leaf, stem, root, inner and outer parts of caudiciform tuber and a pooled quality control (QC) sample was prepared. Samples were quenched and processed as per the Kew Living Collections.

### Metabolite extraction

Methanol (400 μL) and water (400 μL) were sequentially added to 10 mg aliquots of each sample in 2 mL plastic micro-centrifuge tubes, vortexed and rotated for 1 h at room temperature (22 °C). Chloroform (800 μL) was added and the samples vortexed and centrifuged (3 min, 20,000 RCF) to partition extracts into upper (polar) and lower (organic) phases. A 100 μL aliquot of the polar phase was taken into glass vials and succinic-D_4_ acid added as internal standard (10 μL of 1 mg/mL solution). Additionally, a 400 μL aliquot of the non-polar phase, with myristic-D_27_ acid as internal standard, was analysed for the compound atlas. Phases were dried under centrifugal evaporation and stored at −80 °C until analyses. The complete Kew Living collections sample set was extracted on six independent occasions. *D. mexicana* and *D. elephantipes* for the compound atlas were extracted on three independent occasions. Samples received from RBGE were extracted once, due to limited availability of material.

### GC-MS based metabolite profiling

Samples were re-dried for 30 min under centrifugal evaporation before methoxymation and silylation derivatisation via addition of methoxyamine hydrochloride (MeOx; 30 μL, 20 g/L in pyridine) followed by *N*-methyl-*N*-(trimethylsilyl)trifluoroacetamide (MSTFA; 70 μL); incubated (40 °C, 2 h) after addition of each^33^.

Samples (1 μL) were injected into the GC-MS with a split/splitless injector at 290 °C. The injection of samples was made in splitless mode with polar samples of the Kew Living Collections also repeated on a 1:10 split. Metabolites were separated on a DB-5MS + DG 30 m (plus 10 m Duraguard) ×250 μm ×0.25 μm column (J&W Scientific, Folsom, California, US). The GC oven was held for 3 min at 70 °C before ramping at 4 °C/min to 325 °C and held for a min. Helium was the carrier gas at a flowrate of 1.3 mL/min. The interface with the MS was set at 280 °C and MS performed in full scan mode using 70 eV EI + and scanned from 50 to 1000 m/z. Retention time locking to ribitol was used (modified from^44^). A mixture of n-alkanes, ranging from 8 to 32 carbons, was used for retention index external calibration. Kew Living Collections sample sets (6) were run in two batches of three randomised-blocks, two months apart. This approach was used to assess robustness due to the lack of quality control samples. Samples for *D. elephantipes* of the compound atlas were analysed in three blocks within a single batch.

To identify chromatogram components found in the *Dioscorea* profiles, a mass spectral library was constructed from in-house standards, the NIST ‘11 MS library (National Institute of Standards and Technology, USA) and the Golm Metabolome Database (GMD)^45^, with additional manual searches of MassBank^46^, Human Metabolome Database (HMDB)^47^ and the Yeast Metabolome Database (YMDB)^48^. Component peak identification and spectral deconvolution was performed using the Automated Mass Spectral Deconvolution and Identification System (AMDIS v2.71, NIST); using Kovat’s retention indices (RI) and MS for identification using the metabolomics reporting guidelines^49^,^50^. Each compound was assigned a representative ion and response areas were integrated and expressed relative to internal standard.

Linearity was assessed by following the standard extraction procedure on different amounts of material (5, 10 and 20 mg) and the range further enhanced by taking a range of aliquots (10, 20, 50, 100 and 200 μL). Recovery was assessed by expressing measurements from each extract of sample (10 mg) as a percentage of the total following three sequential extractions. All measurements for method development were conducted in triplicate and relative to internal standard. A shikimic acid standard series at 10, 20, 50, 100 and 200 μg from methanolic stock was made on three occasions and analysed as per samples. Ion 255 was chosen to be representative.

### Statistical analysis and visualisation of the data

All data analyses were performed using XLSTAT add-ins (Addinsoft) within Microsoft Excel. Generalised Procrustes Analysis (GPA) was performed using the Commandeur algorithm with 300 simulations. Agglomerative Hierarchical Clustering (AHC) was performed via Spearman dissimilarity with complete linkage. Principal Component Analysis (PCA) was conducted on the Spearman correlation matrix.

Kruskal-Wallis’ one way analysis of variance was performed following normality and variance testing. Monte Carlo permutations (10,000) were used for p-value calculation. Conover-Iman post hoc tests (α = 0.05) were Bonferroni-corrected and selection of most discriminatory metabolites based on the number of groups generated. All univariate tests were two-tailed. More detailed explanations on the choice of statistical tests are provided in Supplementary Methods.

## Additional Information

**How to cite this article**: Price, E. J. *et al*. Metabolite profiling of *Dioscorea* (yam) species reveals underutilised biodiversity and renewable sources for high-value compounds. *Sci. Rep.* **6**, 29136; doi: 10.1038/srep29136 (2016).

## Supplementary Material

Supplementary Information

## Figures and Tables

**Table 1:** *Dioscorea* accessions collected from The Living Collections held at the Royal Botanic Gardens, Kew (http://epic.kew.org/index.htm).

***Dioscorea*****species [in text]**	**Accession**	**Date collected**	**Glasshouse**	**Native habitat**^a^	**Clade**^b^	**Perennating organ type(s)**^c^
*elephantipes* (L’Hér.) Engl. [*D. elephantipes(1)*]	2007–447	21/11/2013	Tropical Nursery	South Africa [SA]	Africa- *Testudinaria*	Caudiciform perennial tuber
*membranacea* Pierre ex Prain & Burkill [*D. membranacea*]	1998–4292	21/11/2013	Tropical Nursery	Asia [Asia]	Stenophora	Rhizome
*elephantipes* (L’Hér.) Engl. [*D. elephantipes(2)*]	2012–54	21/11/2013	Tropical Nursery	South Africa [SA]	Africa-*Testudinaria*	Caudiciform perennial tuber
*sylvatica* Eckl. [*D. sylvatica(1)*]	1963–26704	21/11/2013	Tropical Nursery	South Africa [SA]	Africa-*Testudinaria*	Perennial tuber
*sylvatica* Eckl. [*D. sylvatica(2)*]	1963–26705	21/11/2013	Tropical Nursery	South Africa [SA]	Africa-*Testudinaria*	Perennial tuber
*dumetorum* (Kunth) Pax [*D. dumetorum*]	1984–8405	21/11/2013	Tropical Nursery	Tropical Africa [TA]	Compound leaved-Lasiophyton (Lasiophyton)	Annual tuber & aerial bulbils
*antaly* Jum. & H.Perrier [*D. antaly*]	1998–523	21/11/2013	Tropical Nursery	Madagascar [Mad]	Compound leaved	Annual tuber
*bulbifera* L. [*D. bulbifera(1)*]	1998–533	21/11/2013	Tropical Nursery	Asia [Asia]	Compound leaved (Opsophyton)	Annual tuber & aerial bulbils
*cochleariapiculata* De Wild [*D. cochleari-apiculata(1)*]	1998–2987	21/11/2013	Tropical Nursery	Tropical Africa [TA]	Compound leaved-Lasiophyton	Annual tuber
*pentaphylla* L. [*D. pentaphylla*]	1996–4313	21/11/2013	Tropical Nursery	Asia [Asia]	Compound leaved-Botryosicyos	Annual tuber & aerial bulbils
*sansibarensis* Pax [*D. sansibarensis(1)*]	1998–525	21/11/2013	Tropical Nursery	Madagascar [Mad]	Malagasy	Perennial tuber & aerial bulbils
*altissima* Lam. [*D. altissima*]	2005–1233	21/11/2013	Tropical Nursery	New World [NW]	N.A.^d^	Annual tuber
*cayennensis* Lam. *subsp. rotundata* (Poir.) J. Miège [*D. rotundata*]	1976–1475	25/11/2013	Palm House	Tropical Africa [TA]	(Enantiophyllum)	Annual tuber
*praehensilis* Benth. [*D. praehensilis*]	1960–1002	25/11/2013	Palm House	Tropical Africa [TA]	(Enantiophyllum)	Annual tuber
*preussii* Pax *subsp. preussii* [*D. preussii*]	1968–57006	25/11/2013	Palm House	Tropical Africa [TA]	Enantiophyllum (Macrocarpaea)	Annual tuber
*minutiflora* Engl. [*D. minutiflora*]	1960–1001	25/11/2013	Palm House	Tropical Africa [TA]	Enantiophyllum (Enantiophyllum)	Perennial tuber
*bulbifera* L. [*D. bulbifera(2)*]	1987–1993	25/11/2013	Palm House	Asia [Asia]	Compound leaved (Opsophyton)	Annual tuber & aerial bulbils
*sansibarensis* Pax [*D. sansibarensis(2)*]	1598–543	25/11/2013	Palm House	Madagascar [Mad]	Malagasy	Perennial tuber & aerial bulbils
*composita* Hemsl. [*D. composita(1)*]	1969–11715	25/11/2013	Palm House	New World [NW]	New World I	Perennial tuber
*composita* Hemsl. [*D. composita(2)*]	1978–1830	25/11/2013	Palm House	New World [NW]	New World I	Perennial tuber
*alata* L. [*D. alata*]	1982–1316	25/11/2013	Palm House	Asia (Asia)	Enantiophyllum (Enantiophyllum)	Annual tuber & aerial bulbils
*elephantipes* (L’Hér.) Engl. [*D. elephantipes(3)*]	2012–54	25/11/2013	Princess of Wales	South Africa [SA]	Africa-*Testudinaria*	Caudiciform perennial tuber
*elephantipes* (L’Hér.) Engl. [*D. elephantipes(4)*]	2001–2252	25/11/2013	Princess of Wales	South Africa [SA]	Africa-*Testudinaria*	Caudiciform perennial tuber
*cochleariapiculata* De Wild [*D. cochleari - apiculata(2)*]	1998–2987	25/11/2013	Princess of Wales	Tropical Africa [TA]	Compound leaved-Lasiophyton	Annual tuber
*rockii* Prain & Burkill [*D. rockii*]	1996–4307	25/11/2013	Jodrell	Asia [Asia]	Stenophora	Rhizome
*cochleariapiculata* De Wild [*D. cochleari - apiculata(3)*]	1998–515	25/11/2013	Jodrell	Tropical Africa [TA]	Compound leaved-Lasiophyton	Annual tuber
*glabra* Roxb. [*D. glabra*]	1996–4312	25/11/2013	Jodrell	Asia (Asia)	Enantiophyllum	Annual tuber
*cochleariapiculata* De Wild [*D. cochleari - apiculata(4)*]	1995–1459	25/11/2013	Jodrell	Tropical Africa [TA]	Compound leaved-Lasiophyton	Annual tuber

^a^Habitat used for geographical visualisation of AHC clustering (Fig. 4).

^b^Clades from^38^ and/or(51).

^c^Organ types from^4^ and field experience.

^d^N.A. – Not applicable, where data is unavailable.
