# Supplementary material for: Metabolite profiling of Dioscorea (yam) species reveals underutilised biodiversity and renewable sources for high-value compounds
Source: Sci Rep. 2016 Jul 7;6:29136. doi: 10.1038/srep29136 (PMC4935876; doi:10.1038/srep29136)
Supplement: Supplementary Information [file srep29136-s1.pdf]

Supplementary Information

Metabolite profiling of *Dioscorea* (yam) species reveals underutilised biodiversity and renewable sources for high-value compounds

Elliott J. Price[1,2], Paul Wilkin[2], Viswambharan Sarasan[2] & Paul D. Fraser[1,*]

1. School of Biological Sciences, Royal Holloway University of London, Egham, Surrey, UK, TW20 0EX
2. Royal Botanic Gardens, Kew, Richmond, Surrey, UK, TW20 3AB

Contents:

Supplementary Figure S1. Schematic workflow of developed metabolite profiling platform applied to *Dioscorea* species. .

Supplementary Table S2. Linearity of representative polar metabolites in *Dioscorea* detected by GC-MS platform developed.

Supplementary Table S3. Recovery of representative polar metabolites in successive extractions of *Dioscorea* leaf material.

Supplementary Table S4. Mean abundance of identified metabolites recorded in *Dioscorea* material of the Kew Living Collections via the devised GC-MS platform.

Supplementary Figure S5. Consensus GPA bi-plots on the polar fraction of leaf extracts from the 28 *Dioscorea* accessions.

Supplementary Figure S6. Heat-map showing qualitative and quantitative differences in metabolic compositions between accessions, extended from Figure 2.

Supplementary Figure S7. PCA plots following metabolite profiling on different sections of *D. elephantipes*.

Supplementary Methods. Further explanation of statistical analyses.

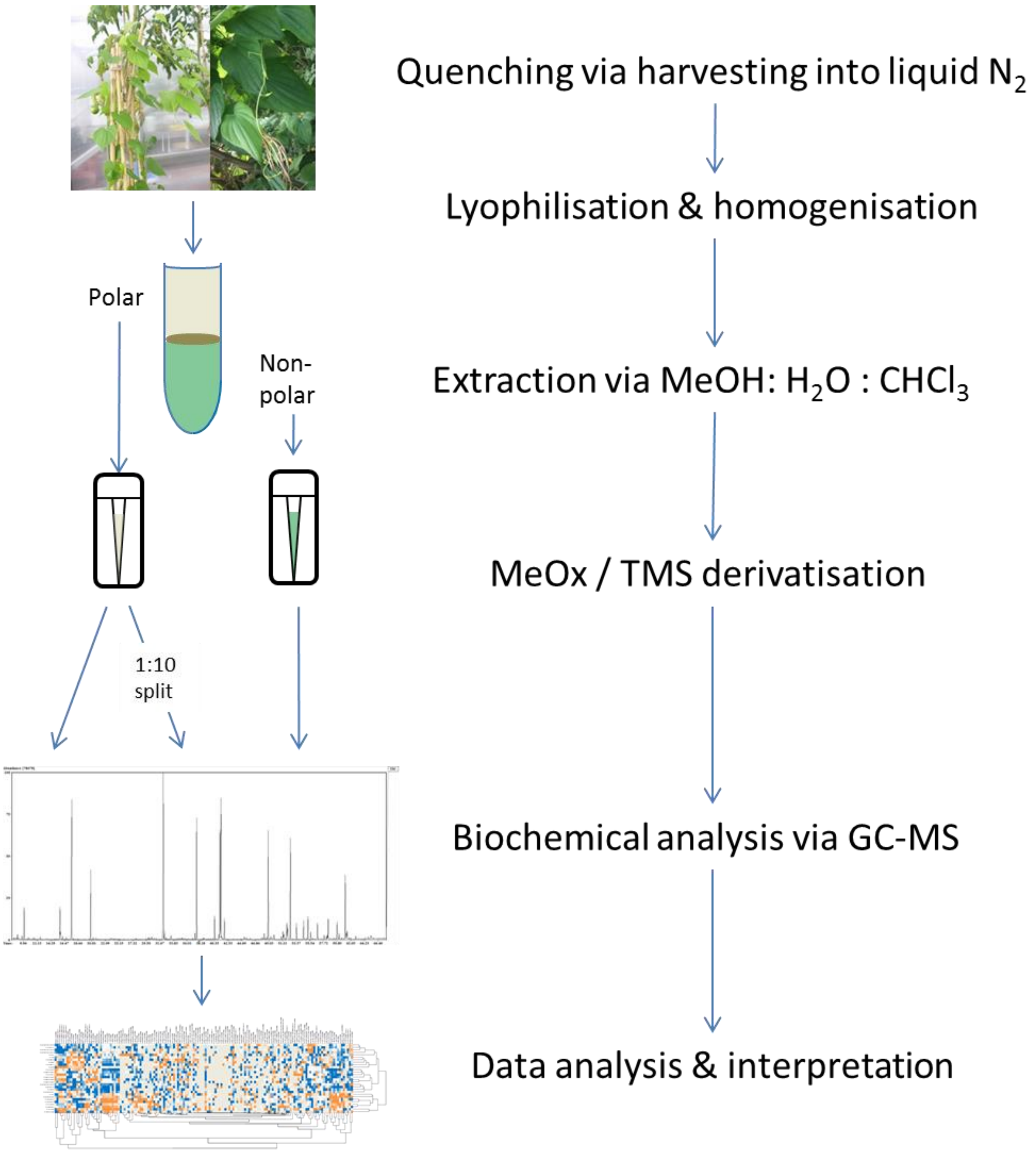

Supplementary Figure S1. Schematic workflow of developed metabolite profiling platform applied to *Dioscorea* species.

Supplementary Table S2. Linearity of representative polar metabolites in *Dioscorea* detected by GC-MS platform developed.

Metabolite	R^2	Range*	Equation
Lactic acid (2TMS)	0.9551	1000-4000	y = 6E-06x - 0.0029
L-Alanine (2TMS)	0.9831	50-4000	y = 0.0001x - 0.0226
Glycine (2TMS)	0.9668	500-4000	y = 2E-06x - 0.001
Malonic acid (2TMS)	0.9888	1000-4000	y = 0.0004x - 0.0161
L-Valine (2TMS)	0.9927	200-4000	y = 3E-05x - 0.0093
Ethanolamine (3TMS)	0.9879	50-4000	y = 0.0007x - 0.0784
L-Leucine (2TMS)	0.9801	500-4000	y = 2E-05x - 0.0102
Phosphate (3TMS)	0.9864	50-4000	y = 0.0028x - 0.3814
Isoleucine (2TMS)	0.9975	1000-4000	y = 3E-05x - 0.0087
L-Proline (2TMS)	0.9988	1000-4000	y = 2E-05x - 0.0072
Glyceric acid (3TMS)	0.9405	500-4000	y = 2E-05x - 0.011
Fumaric acid (2TMS)	0.9243	50-4000	y = 2E-05x - 0.0052
L-Serine (3TMS)	0.9824	50-4000	y = 0.0017x - 0.3024
L-Threonine (3TMS)	0.9942	50-4000	y = 0.0003x - 0.042
Malic acid (3TMS)	0.9806	50-4000	y = 0.002x - 0.4263
Threitol (4TMS)	0.99	50-2000	y = 0.0007x - 0.0501
Pyroglutamic acid (2TMS)	0.9995	50-4000	y = 0.0005x + 0.0243
L-Aspartic acid (3TMS)	0.9947	50-1000	y = 0.0003x - 0.0373
GABA (3TMS)	0.9919	50-4000	y = 0.0001x - 0.0244
Xylulose (4TMS) isomer 2	0.9509	50-4000	y = 0.0032x - 0.8591
Ornithine (3TMS) isomer 1	0.9983	1000-4000	y = 0.0003x - 0.1029
Phenylalanine (2TMS)	0.9886	50-4000	y = 8E-05x - 0.0164
L-Asparagine (2TMS)	0.9957	50-4000	y = 1E-04x - 0.0121
Allantoin derivative 1	0.9749	50-4000	y = 0.0014x - 0.3783
Xylitol (5TMS)	0.9972	200-4000	y = 8E-05x - 0.0109
Ribitol (5TMS)	0.9905	500-4000	y = 7E-05x - 0.0221
Putrescine (4TMS)	0.9971	1000-4000	y = 2E-05x - 0.0074
Ornithine (3TMS) isomer 2	0.9418	50-4000	y = 6E-05x - 0.018
Allantoin derivative 2	0.9766	50-4000	y = 0.0006x + 0.0548
Methylfructoside (4TMS)	0.749	50-4000	y = 0.0003x - 0.1169
Fructose (5TMS) isomer 1	0.9929	50-1000	y = 0.0177x - 8.0905
Ornithine (4TMS)	0.9906	50-2000	y = 0.0016x - 0.1355
Fructose (5TMS) isomer 2	0.9993	50-1000	y = 0.0067x - 3.1931
Citric acid (4TMS)	0.9952	200-2000	y = 0.0008x - 0.2785
Arginine [-NH3] (3TMS)	0.9728	50-4000	y = 0.0004x - 0.1047
Estra-1,3,5(10)-trien-6-one, (16α,17β)- (3TMS)	0.9909	100-2000	y = 0.0002x - 0.0694
Fructose (1MEOX 5TMS) isomer 1	0.9909	50-500	y = 0.0123x + 3.7382
Fructose (1MEOX 5TMS) isomer 2	0.9946	50-500	y = 0.0086x - 4.0927
Glucose (1MEOX 5TMS) isomer 1	0.9846	50-500	y = 0.0024x - 1.1549
Glucose (1MEOX 5TMS) isomer 2	0.9792	50-500	y = 0.0031x + 0.0165
L-Lysine (4TMS)	0.996	200-1000	y = 0.0003x - 0.0787
Mannitol (6TMS) isomer 1	0.9949	200-4000	y = 6E-05x - 0.0049
L-Tyrosine (3TMS)	0.9501	1000-4000	y = 1E-05x - 0.0065
Mannitol (6TMS) isomer 2	0.9917	100-2000	y = 0.0001x - 0.0503
Glucopyranose (5TMS)	0.9785	200-1000	y = 0.0288x - 13.128
Gluconic acid (6TMS)	0.9991	1000-4000	y = 5E-05x - 0.0517
Inositol, scyllo (6TMS)	0.9541	50-500	y = 0.0046x - 1.1565
Catechollactate (4TMS)	0.9834	500-4000	y = 2E-05x + 0.0024
Dopamine (3TMS)	0.9949	50-2000	y = 0.0001x - 0.0145
Sedoheptulose (1MEOX 6TMS)	0.9608	50-2000	y = 0.0002x - 0.0237
Tryptophan (2TMS)	0.995	1000-4000	y = 6E-05x - 0.0644
Inositol-2-phopsphate , myo- (7TMS)	0.9995	1000-4000	y = 2E-05x - 0.0076
Sucrose (6TMS)	0.9879	50-500	y = 0.0283x + 1.4943
Melibiose (8TMS) isomer 1	0.9858	1000-4000	y = 6E-05x - 0.0147
Similar to Caffeic acid (3TMS) 1	0.988	1000-4000	y = 3E-05x - 0.0115
Similar to Sucrose (8TMS) 2	0.8857	50-4000	y = 1E-05x - 0.0043
Similar to Caffeic acid (3TMS) 3	0.9973	400-4000	y = 0.0003x - 0.0808

*Range = mg of material * µL aliquot taken

Supplementary Table S3. Recovery* of representative polar metabolites in successive extractions of *Dioscorea* leaf material.

	Extraction number		
**Metabolite**	**1**	**2**	**3**
Lactic acid (2TMS)	21.97	54.61	23.42
L-Alanine (2TMS)	100.00	0.00	0.00
Hydroxylamine (3TMS)	36.57	56.17	7.26
Malonic acid (2TMS)	100.00	0.00	0.00
L-Valine (2TMS)	91.05	8.95	0.00
Serine (2TMS)	84.29	15.71	0.00
Ethanolamine (3TMS)	83.08	16.92	0.00
L-Leucine (2TMS)	100.00	0.00	0.00
Glycerol (3TMS)	47.45	27.49	25.06
Phosphate (3TMS)	83.35	15.27	1.38
Isoleucine (2TMS)	94.22	5.78	0.00
L-Proline (2TMS)	100.00	0.00	0.00
Glycine (3TMS)	100.00	0.00	0.00
Succinic acid (2TMS)	31.26	33.20	35.53
Fumaric acid (2TMS)	100.00	0.00	0.00
L- Aspartic acid (2TMS)	100.00	0.00	0.00
Malic acid (3TMS)	97.37	2.63	0.00
Erythritol (4TMS)	100.00	0.00	0.00
GABA (3TMS)	100.00	0.00	0.00
Threonic acid (4TMS)	100.00	0.00	0.00
Xylulose (4TMS) isomer 1	100.00	0.00	0.00
Xylulose (4TMS) isomer 2	100.00	0.00	0.00
Xylitol (5TMS)	100.00	0.00	0.00
Ribitol (5TMS)	100.00	0.00	0.00
Citric acid (4TMS)	100.00	0.00	0.00
Isocitric acid (4TMS)	100.00	0.00	0.00
Fructose (1MEOX 5TMS) isomer 1	65.97	18.15	15.89
Fructose (1MEOX 5TMS) isomer 2	66.67	33.33	0.00
Galactose (1MEOX 5TMS) isomer 1	100.00	0.00	0.00
Glucose (1MEOX 5TMS) isomer 1	66.43	33.57	0.00
Galactose (1MEOX 5TMS) isomer 2	100.00	0.00	0.00
Glucose (1MEOX 5TMS) isomer 2	65.12	15.82	19.06
Mannitol (6TMS) isomer 1	100.00	0.00	0.00
Mannitol (6TMS) isomer 2	100.00	0.00	0.00
Inositol, scyllo (6TMS)	81.49	13.99	4.52
Sedoheptulose (1MEOX 6TMS)	100.00	0.00	0.00
Sucrose (8TMS)	78.82	18.05	3.13
Maltose (1MEOX 8TMS)	100.00	0.00	0.00
Melibiose (8TMS) isomer 1	85.82	14.18	0.00

*Mean (n=3) recovery, expressed as a percentage of total from all sucessive extracts.

Supplementary Table S4. Mean abundance* (n=6) of identified** metabolites recorded in *Dioscorea* material of the Kew Living Collections via the devised GC-MS platform.

						Accession from Kew Living Collections																											
Metabolite	RT	Confirmation status		Reference ion	Match	*D. elephantipes(1)*	*D. membranacea*	*D. elephantipes(2)*	*D. sylvatica(1)*	*D. sylvatica(2)*	*D. dumetorum*	*D. antaly*	*D. bulbifera(1)*	*D. cochleari-apiculata(1)*	*D. pentaphylla*	*D. sansibarensis(1)*	*D. altissima*	*D. rotundata*	*D. praehensilis*	*D. preussii*	*D. minutiflora*	*D. bulbifera(2)*	*D. sansibarensis(2)*	*D. composita(1)*	*D. composita(2)*	*D. alata*	*D. elephantipes(3)*	*D. elephantipes(4)*	*D. cochleari-apiculata(2)*	*D. rockii*	*D. cochleari-apiculata(3)*	*D. glabra*	*D. cochleari-apiculata(4)*
Pyruvic acid (1MEOX 1TMS)	9.359	Standard	1	174		2.75E-04	N.D.	N.D.	3.65E-04	8.74E-04	N.D.	N.D.	4.22E-05	5.51E-04	N.D.	1.84E-04	N.D.	6.77E-04	2.90E-04	N.D.	6.37E-03	2.40E-03	N.D.	N.D.	N.D.	N.D.	N.D.	N.D.	3.77E-04	8.87E-04	1.54E-03	6.37E-04	2.74E-03
Lactic acid (2TMS)	9.4242	Standard	1	117		8.48E-03	1.37E-02	6.45E-03	2.19E-02	8.34E-03	1.96E-03	2.15E-03	N.D.	9.99E-03	4.95E-03	5.13E-03	N.D.	1.22E-02	2.82E-02	1.26E-02	3.59E-03	1.90E-02	8.79E-03	4.25E-03	7.64E-03	1.46E-02	6.73E-03	5.29E-03	8.44E-03	1.27E-01	9.44E-03	1.93E-01	4.79E-02
L-Alanine (2TMS)	10.5702	Standard	1	116		9.84E-03	9.59E-02	2.02E-02	5.18E-03	3.94E-03	1.47E-02	1.04E-02	5.68E-03	5.92E-02	3.41E-03	1.05E-02	4.34E-03	7.41E-02	7.77E-02	1.62E-02	6.88E-02	3.19E-02	7.36E-02	1.21E-01	8.55E-02	4.09E-02	9.24E-03	2.13E-03	8.88E-03	9.16E-02	1.94E-02	2.10E-02	2.29E-02
Glycine (2TMS)	10.9753	Standard	1	102		7.72E-05	4.11E-03	1.74E-04	N.D.	N.D.	6.88E-04	N.D.	N.D.	1.99E-03	N.D.	N.D.	N.D.	2.84E-03	5.15E-03	N.D.	3.34E-03	N.D.	5.47E-03	9.44E-03	4.20E-03	N.D.	N.D.	N.D.	N.D.	2.25E-03	2.90E-03	9.12E-04	2.60E-03
Malonic acid (2TMS)	13.7373	Database (NIST & GMD)	2	147	879	N.D.	9.37E-03	N.D.	N.D.	N.D.	N.D.	6.39E-03	3.88E-02	9.81E-05	N.D.	1.23E-04	1.05E-03	1.01E-02	3.07E-03	N.D.	6.30E-04	1.71E-04	1.16E-03	1.08E-03	2.22E-03	3.89E-04	N.D.	N.D.	1.04E-03	2.24E-02	N.D.	3.32E-04	N.D.
L-Valine (2TMS)	14.0614	Standard	1	144		2.00E-02	1.70E-01	4.67E-02	2.22E-02	9.35E-03	3.21E-02	2.06E-02	6.38E-03	6.75E-02	6.41E-03	1.04E-02	2.21E-03	2.04E-01	1.66E-01	1.63E-02	1.18E-01	2.14E-02	1.10E-01	3.77E-01	1.96E-01	3.63E-02	3.26E-02	4.27E-03	1.32E-02	9.92E-02	3.63E-02	4.80E-02	3.10E-02
Serine (2TMS)	15.4629	Standard	1	116		3.08E-03	1.20E-02	8.03E-03	5.53E-03	3.40E-03	5.61E-03	9.22E-04	9.04E-03	4.96E-03	1.13E-04	2.61E-02	3.84E-02	7.94E-03	3.14E-02	8.76E-03	3.11E-02	1.11E-02	6.59E-02	2.71E-02	1.46E-02	1.42E-02	9.04E-03	9.59E-04	9.82E-03	1.62E-02	2.96E-03	2.83E-02	1.69E-03
Ethanolamine (3TMS)	15.7362	Database (NIST & GMD)	2	174	950	7.11E-02	1.12E-01	4.26E-01	2.98E-02	1.08E-02	7.14E-02	1.60E-01	5.09E-02	1.11E-01	3.18E-02	7.23E-02	2.54E-02	4.62E-01	1.40E-01	6.81E-02	1.58E-01	7.55E-02	1.69E-01	1.23E-01	8.82E-02	1.19E-01	3.56E-01	4.72E-02	4.54E-02	1.15E-01	1.39E-01	1.17E-01	1.03E-01
L-Leucine (2TMS)	15.791	Standard	1	158		1.76E-02	1.71E-01	3.74E-02	1.43E-02	6.21E-03	2.31E-02	1.69E-02	2.71E-03	2.47E-02	4.51E-03	4.73E-03	2.14E-04	2.70E-01	1.43E-01	1.11E-02	7.84E-02	1.26E-02	8.88E-02	2.44E-01	1.34E-01	2.30E-02	2.21E-02	3.80E-03	8.61E-03	7.47E-02	4.97E-02	4.99E-02	5.23E-02
Phosphate (3TMS)	15.9743	Standard	1	299		5.17E-01	2.75E-01	6.58E-01	7.36E-01	4.76E-01	6.09E-01	6.83E-01	2.04E-01	1.84E-01	7.53E-01	7.69E-01	2.24E+00	1.05E-01	1.10E+00	3.44E-01	9.91E-01	2.54E-01	9.24E-01	9.37E-01	7.30E-01	1.96E-01	3.62E-01	1.82E-01	8.50E-01	1.30E+00	1.45E-01	1.36E-01	3.05E-01
Glycerol (3TMS)	16.0468	Standard	1	205		2.20E-01	2.22E-01	2.15E-01	2.68E-02	1.71E-02	1.57E-01	7.02E-02	1.50E-02	7.23E-02	1.53E-02	8.98E-02	7.74E-02	1.38E-01	3.14E-01	3.12E-02	3.56E-01	8.16E-02	5.88E-01	1.79E-01	1.38E-01	8.76E-02	9.81E-02	7.60E-02	1.00E-01	2.95E-01	1.39E-01	2.96E-01	1.62E-01
Isoleucine (2TMS)	16.4676	Standard	1	158		1.34E-02	1.33E-01	3.59E-02	2.13E-02	8.42E-03	2.16E-02	2.28E-02	4.05E-03	3.56E-02	4.71E-03	5.67E-03	1.61E-03	1.45E-01	1.32E-01	9.70E-03	8.87E-02	1.16E-02	8.49E-02	3.22E-01	1.68E-01	1.97E-02	2.61E-02	3.12E-03	1.09E-02	5.67E-02	2.80E-02	3.37E-02	2.10E-02
Nicotinic acid (1TMS)	16.6341	Standard	1	180		1.97E-03	3.16E-03	9.17E-04	6.49E-04	1.42E-03	1.26E-02	1.36E-03	1.22E-03	8.54E-03	1.33E-03	3.06E-03	1.66E-03	8.14E-03	5.99E-03	1.69E-02	8.14E-03	1.32E-02	1.06E-02	3.22E-03	3.19E-03	1.09E-02	2.41E-03	1.03E-03	1.25E-03	5.69E-03	5.17E-02	1.08E-02	2.47E-02
L-Proline (2TMS)	16.6351	Standard	1	142		4.10E-03	4.27E-02	8.55E-03	2.77E-03	8.55E-04	1.41E-02	1.06E-02	2.11E-03	8.95E-03	9.36E-04	2.87E-03	1.65E-03	1.36E-01	6.23E-02	6.63E-03	2.39E-02	5.10E-03	1.91E-01	9.96E-02	6.64E-02	1.57E-02	7.29E-03	7.12E-04	2.54E-03	2.13E-02	2.02E-02	2.45E-02	1.66E-02
Threonine (2TMS)	16.6474	Standard	1	117		N.D.	2.40E-03	6.15E-04	2.01E-03	1.57E-03	N.D.	7.00E-04	N.D.	N.D.	N.D.	N.D.	1.12E-04	1.08E-02	4.24E-03	N.D.	4.42E-03	N.D.	2.69E-03	N.D.	1.03E-03	N.D.	N.D.	7.09E-04	N.D.	8.57E-04	N.D.	6.32E-03	N.D.
Maleic acid (2TMS)	16.9328	Standard	1	245		2.09E-02	3.06E-01	1.08E-02	1.79E-02	2.45E-02	4.65E-03	5.13E-02	8.01E-02	1.17E-01	1.21E-02	4.09E-02	1.65E-02	7.10E-02	3.28E-03	1.28E-01	3.44E-03	3.83E-02	N.D.	N.D.	3.11E-04	8.21E-02	N.D.	2.53E-02	9.26E-03	8.23E-03	3.02E-03	1.21E-01	7.13E-03
Succinic acid (2TMS)	17.3484	Standard	1	247		5.53E-03	2.44E-03	N.D.	2.50E-03	4.34E-03	N.D.	N.D.	3.64E-03	N.D.	N.D.	1.60E-02	N.D.	N.D.	9.40E-03	N.D.	6.23E-04	N.D.	1.24E-02	N.D.	N.D.	3.60E-03	N.D.	1.68E-04	N.D.	N.D.	N.D.	N.D.	N.D.
Picolinic acid (1TMS)	17.5452	Standard	1	180		5.10E-05	2.95E-04	1.68E-04	5.56E-05	1.57E-04	2.02E-04	9.06E-05	2.66E-04	1.36E-04	4.36E-04	1.64E-04	3.00E-04	4.42E-04	4.18E-04	3.33E-04	3.42E-04	1.39E-04	3.49E-04	5.40E-04	3.25E-04	3.49E-04	3.16E-05	5.91E-05	1.51E-04	5.02E-04	2.10E-04	6.47E-04	1.13E-04
Glyceric acid (3TMS)	17.8092	Standard	1	292		4.67E-02	1.80E-01	4.12E-02	2.99E-02	2.98E-02	7.00E-02	1.67E-01	5.86E-02	1.26E-01	1.01E-02	1.48E-01	1.87E-02	7.82E-02	1.96E-02	9.76E-02	8.41E-02	7.03E-02	3.00E-01	1.10E-01	3.27E-02	2.64E-01	3.76E-02	9.36E-02	1.84E-01	7.85E-02	1.22E-01	9.33E-02	1.59E-01
Itaconic acid (2TMS)	18.2112	Standard	1	215		8.74E-03	2.23E-02	9.65E-03	3.04E-03	1.51E-03	6.68E-03	3.78E-02	1.15E-02	1.63E-02	3.04E-03	9.02E-03	5.56E-03	4.13E-03	8.52E-03	3.10E-02	4.30E-02	2.13E-02	5.85E-03	1.00E-02	1.10E-02	1.41E-02	7.10E-04	7.08E-03	9.95E-03	3.99E-02	3.98E-03	2.35E-02	1.36E-02
Fumaric acid (2TMS)	18.5332	Standard	1	245		N.D.	2.53E-01	N.D.	N.D.	3.22E-02	N.D.	1.75E-02	4.36E-02	5.65E-02	N.D.	4.24E-02	5.91E-03	4.37E-02	2.76E-03	1.08E-01	2.39E-03	1.55E-02	5.38E-02	8.66E-03	N.D.	6.69E-02	N.D.	3.20E-03	N.D.	8.20E-04	N.D.	4.29E-02	N.D.
L-Serine (3TMS)	18.7878	Standard	1	204		4.03E-02	2.42E-01	8.50E-02	2.96E-02	1.37E-02	6.31E-02	1.26E-01	1.12E-01	2.63E-01	1.38E-02	2.01E-01	9.86E-02	3.28E-01	3.90E-01	1.26E-01	3.63E-01	1.39E-01	1.14E+00	5.91E-01	4.98E-01	1.84E-01	2.16E-01	1.87E-02	1.12E-01	4.59E-01	1.01E-01	2.08E-01	1.37E-01
L-Threonine (3TMS)	19.6052	Standard	1	218		2.96E-02	1.63E-01	6.44E-02	2.75E-02	1.73E-02	4.68E-02	7.12E-02	2.02E-02	1.19E-01	8.76E-03	3.56E-02	3.23E-02	2.66E-01	2.35E-01	2.90E-02	1.60E-01	3.41E-02	4.04E-01	4.58E-01	2.77E-01	5.17E-02	6.00E-02	1.03E-02	3.12E-02	1.69E-01	5.74E-02	7.20E-02	7.10E-02
Mesaconic acid (2TMS)	20.1052	Standard	1	184		9.89E-04	1.37E-03	8.39E-04	N.D.	N.D.	4.80E-04	4.01E-03	5.52E-04	1.40E-03	9.01E-05	8.10E-04	1.76E-04	8.94E-05	8.03E-04	4.19E-03	5.38E-03	2.87E-03	5.15E-04	9.67E-04	1.28E-03	6.36E-04	N.D.	6.25E-04	8.85E-04	4.06E-03	1.34E-04	1.85E-03	2.39E-03
L-Aspartic acid (2TMS)	20.7659	Standard	1	116		N.D.	2.10E-02	N.D.	N.D.	N.D.	2.21E-03	2.32E-03	1.73E-02	N.D.	7.10E-03	1.87E-02	1.51E-02	1.50E-02	3.04E-02	3.27E-02	2.29E-02	2.93E-02	3.69E-02	3.33E-02	1.55E-02	3.90E-02	1.52E-03	6.91E-03	2.85E-02	4.03E-02	2.93E-03	1.56E-02	2.21E-03
Ornithine-1,5-lactam (2TMS)	21.7013	Database (NIST & GMD)	2	128	715	6.17E-04	5.95E-03	6.47E-03	N.D.	N.D.	1.58E-03	4.95E-01	N.D.	7.65E-02	N.D.	N.D.	7.55E-04	2.20E-02	1.03E-02	N.D.	4.02E-03	N.D.	2.07E-02	4.04E-02	1.49E-01	2.74E-04	6.45E-03	N.D.	N.D.	1.11E-01	N.D.	6.34E-04	2.95E-03
Similar to L-Citrulline (3TMS)	22.0674	Database (NIST)	3	142	728	N.D.	N.D.	N.D.	N.D.	N.D.	N.D.	8.15E-04	N.D.	N.D.	N.D.	N.D.	N.D.	N.D.	N.D.	N.D.	N.D.	N.D.	N.D.	N.D.	N.D.	N.D.	N.D.	N.D.	N.D.	N.D.	N.D.	N.D.	N.D.
Citramalic acid (3TMS)	22.16703	Database (NIST & GMD)	2	246	936	1.32E-02	7.66E-04	3.02E-03	2.97E-03	4.01E-03	N.D.	3.55E-03	1.97E-03	N.D.	N.D.	1.87E-03	2.39E-03	8.19E-04	N.D.	2.35E-03	8.24E-04	4.39E-04	2.13E-03	N.D.	N.D.	2.04E-04	N.D.	9.52E-03	N.D.	5.97E-04	N.D.	9.21E-04	N.D.
Arabino-Hexos-2-ulose (4TMS)	22.5093	Database (NIST)	3	234	883	3.68E-03	3.05E-02	1.30E-03	7.04E-03	6.64E-03	5.59E-02	N.D.	1.46E-03	5.77E-02	N.D.	1.04E-03	6.38E-03	6.87E-04	2.72E-03	3.54E-03	6.68E-03	1.99E-03	N.D.	6.20E-03	2.18E-03	1.89E-03	3.92E-04	5.71E-03	4.55E-04	1.25E-01	9.35E-02	3.29E-03	1.50E-01
Malic acid (3TMS)	22.8599	Standard	1	233		2.69E-01	4.18E+00	1.56E-01	6.44E-01	1.69E+00	4.17E-01	1.24E+00	1.96E+00	2.76E+00	1.59E-01	1.18E+00	3.25E-01	2.27E+00	6.53E-01	3.01E+00	5.26E-01	1.06E+00	1.74E+00	2.73E-01	7.29E-02	2.37E+00	4.17E-02	7.04E-01	1.05E-01	9.53E-01	2.81E-01	1.56E+00	2.53E-01
Threitol (4TMS)	23.1639	Standard	1	217		1.38E-01	2.70E+00	8.97E-02	3.39E-01	7.97E-01	2.47E-01	6.00E-01	9.68E-01	1.54E+00	8.87E-02	5.41E-01	1.53E-01	1.08E+00	3.39E-01	1.53E+00	2.85E-01	5.21E-01	8.29E-01	1.33E-01	7.83E-02	1.21E+00	4.29E-02	3.53E-01	4.80E-02	4.76E-01	2.12E-01	6.22E-01	1.40E-01
Pyroglutamic acid (1TMS)	23.2406	Standard	1	84		N.D.	1.46E-04	N.D.	N.D.	N.D.	N.D.	N.D.	N.D.	N.D.	N.D.	N.D.	N.D.	N.D.	N.D.	N.D.	N.D.	N.D.	1.04E-03	N.D.	N.D.	N.D.	N.D.	N.D.	N.D.	N.D.	N.D.	1.28E-03	N.D.
Salicylic acid (2TMS)	23.2543	Standard	1	267		6.28E-04	1.22E-03	1.73E-03	3.52E-04	5.43E-05	N.D.	2.73E-04	1.50E-04	1.27E-04	3.10E-04	7.87E-05	1.80E-04	2.09E-04	6.53E-03	N.D.	8.00E-04	2.67E-04	5.61E-05	1.08E-04	1.12E-03	8.52E-04	7.46E-04	6.95E-04	6.35E-04	4.01E-04	1.55E-03	3.66E-03	1.47E-04
Erythritol (4TMS)	23.3848	Standard	1	217		7.10E-02	2.43E-02	4.68E-02	4.70E-02	7.48E-02	3.71E-02	1.19E-01	4.69E-02	1.37E-02	1.81E-02	1.07E-02	1.51E-02	1.95E-02	2.17E-02	1.34E-02	3.33E-02	3.22E-02	2.63E-02	5.40E-02	7.70E-02	2.77E-02	7.94E-02	7.13E-02	5.99E-02	4.18E-03	7.29E-03	3.90E-02	1.29E-02
L-Methionine (2TMS)	23.6206	Standard	1	176		1.49E-03	1.56E-02	4.10E-03	1.35E-03	2.18E-04	6.61E-03	2.30E-03	2.05E-04	7.24E-03	3.20E-04	1.10E-04	N.D.	3.08E-02	1.64E-02	8.77E-04	4.96E-03	9.68E-04	2.14E-02	4.05E-02	2.68E-02	2.92E-03	3.14E-03	6.22E-05	1.82E-04	1.68E-02	5.06E-03	5.80E-03	7.77E-03
Pyroglutamic acid (2TMS)	23.7741	Standard	1	156		5.00E-02	8.65E-02	9.69E-02	8.81E-02	6.49E-02	6.69E-02	1.55E-01	2.32E-01	1.34E-01	9.77E-02	3.20E-01	1.28E-01	2.50E-01	4.56E-01	3.81E-01	2.25E-01	2.82E-01	3.13E-01	8.38E-02	1.82E-01	3.44E-01	1.19E-01	4.78E-02	2.57E-01	7.46E-01	1.26E-01	2.62E-01	2.37E-01
Cytosine (2TMS)	23.8079	Standard	1	254		N.D.	2.82E-04	2.57E-04	N.D.	N.D.	N.D.	N.D.	N.D.	N.D.	N.D.	6.39E-04	N.D.	1.82E-03	1.65E-03	3.06E-04	3.23E-04	1.35E-04	7.50E-04	6.40E-04	1.28E-03	3.21E-04	N.D.	N.D.	N.D.	5.56E-04	N.D.	N.D.	N.D.
L-Aspartic acid (3TMS)	23.7363	Standard	1	232		8.52E-03	9.77E-02	3.58E-02	4.35E-03	1.76E-03	3.85E-02	1.26E-01	5.33E-02	5.53E-02	2.50E-02	1.51E-01	7.92E-02	1.50E-01	3.38E-01	2.24E-01	1.97E-01	1.52E-01	3.42E-01	4.05E-01	2.18E-01	2.10E-01	1.62E-02	7.44E-03	1.53E-01	2.14E-01	2.83E-02	7.22E-02	8.91E-02
GABA (3TMS)	24.0623	Standard	1	174		1.37E-01	6.23E-01	2.36E-01	2.59E-02	5.38E-02	1.47E-01	4.80E-02	2.07E-02	7.41E-02	4.09E-02	8.74E-02	5.03E-02	1.79E-01	3.00E-01	1.58E-01	5.88E-01	6.99E-02	4.67E-01	1.08E+00	6.30E-01	8.38E-02	1.34E-01	6.11E-02	2.27E-02	1.39E-01	1.55E-01	1.57E-01	9.34E-02
Phenylalanine (1TMS)	24.4139	Standard	1	120		N.D.	5.13E-03	N.D.	N.D.	N.D.	3.52E-03	N.D.	N.D.	1.28E-03	2.15E-04	N.D.	N.D.	N.D.	N.D.	N.D.	N.D.	N.D.	N.D.	1.87E-02	1.55E-02	N.D.	2.82E-03	N.D.	N.D.	1.31E-02	5.00E-03	N.D.	2.48E-03
2-Deoxyribose (1MEOX 3TMS)	24.5434	Database (NIST & GMD)	2	147	719	N.D.	1.31E-03	N.D.	N.D.	N.D.	5.05E-03	N.D.	N.D.	2.45E-03	1.69E-03	6.00E-03	3.53E-04	N.D.	N.D.	N.D.	N.D.	2.92E-03	2.69E-03	N.D.	N.D.	6.58E-03	N.D.	N.D.	N.D.	N.D.	3.32E-03	2.96E-03	8.25E-03
Norvaline (3TMS)	24.6359	Database (NIST)	3	232	728	N.D.	N.D.	N.D.	N.D.	N.D.	N.D.	N.D.	N.D.	N.D.	N.D.	N.D.	N.D.	N.D.	N.D.	N.D.	N.D.	N.D.	N.D.	N.D.	N.D.	N.D.	N.D.	N.D.	N.D.	3.96E-03	N.D.	N.D.	N.D.
L-Cysteine (3TMS)	24.7316	Standard	1	220		2.37E-04	2.05E-02	2.29E-03	N.D.	N.D.	4.39E-03	1.93E-03	N.D.	7.15E-03	1.21E-03	2.84E-03	N.D.	3.43E-03	8.85E-03	3.58E-03	4.63E-03	1.59E-03	2.50E-03	3.44E-02	2.19E-02	2.24E-03	1.79E-03	N.D.	4.90E-04	1.20E-02	4.20E-03	3.86E-03	3.20E-03
Threonic acid (4TMS)	24.9568	Standard	1	292		9.53E-03	7.92E-02	1.03E-02	2.24E-02	2.23E-02	7.83E-02	3.78E-03	2.99E-02	1.42E-01	1.03E-03	7.89E-03	3.31E-02	3.76E-02	1.59E-02	2.59E-02	1.84E-02	1.74E-02	1.41E-02	2.04E-02	5.74E-03	5.61E-02	6.17E-03	3.37E-02	4.45E-03	1.62E-01	1.64E-01	3.11E-02	2.24E-01
3-Hydroxybenzoic acid (2TMS)	25.0643	Database (NIST & Massbank)	2	267	944	4.32E-03	7.11E-03	5.24E-03	6.42E-03	4.67E-03	2.80E-03	1.20E-02	1.54E-02	4.81E-03	4.54E-03	5.32E-03	3.47E-03	6.33E-03	7.53E-03	2.51E-02	7.50E-03	9.82E-03	5.49E-03	5.50E-03	3.42E-03	6.39E-03	6.90E-03	4.16E-03	6.03E-03	3.08E-03	1.90E-03	5.17E-03	6.85E-03
Tyrosol (2TMS)	25.1831	Database (NIST & YMDB)	2	179	861	N.D.	N.D.	3.64E-03	N.D.	N.D.	N.D.	8.48E-04	N.D.	N.D.	N.D.	N.D.	N.D.	N.D.	N.D.	N.D.	N.D.	N.D.	N.D.	5.10E-04	1.84E-03	N.D.	N.D.	3.26E-04	N.D.	N.D.	N.D.	N.D.	N.D.
2-Isopropylmalic acid (3TMS)	25.2284	Database (NIST & GMD)	2	275	702	N.D.	N.D.	N.D.	N.D.	N.D.	N.D.	N.D.	N.D.	N.D.	N.D.	N.D.	N.D.	7.18E-03	N.D.	N.D.	N.D.	N.D.	N.D.	N.D.	N.D.	N.D.	N.D.	N.D.	N.D.	1.32E-03	N.D.	N.D.	N.D.
Xylulose (4TMS) isomer 1	25.3134	Standard	1	306		1.06E+00	5.86E-03	9.23E-02	3.17E+00	2.08E+00	1.40E-03	9.17E-01	1.83E+00	1.47E-02	1.32E-03	3.90E-01	1.13E-01	2.69E+00	4.43E-01	2.69E+00	1.13E-01	7.12E-01	7.10E-02	3.51E-04	2.29E-04	1.48E+00	1.76E-01	8.86E-01	4.54E-01	3.02E-02	1.05E-02	4.23E+00	6.00E-03
2-Ketoglutaric acid (1MEOX 2TMS)	25.4461	Standard	1	198		1.20E-02	1.70E-04	2.60E-04	5.60E-02	7.59E-02	2.62E-04	N.D.	6.02E-02	2.70E-03	1.71E-03	2.92E-04	2.71E-01	N.D.	3.93E-04	4.71E-04	3.74E-04	3.84E-03	1.26E-03	N.D.	N.D.	3.74E-04	N.D.	1.39E-02	N.D.	7.53E-03	6.35E-03	N.D.	N.D.
Tropic acid (2TMS)	25.467	Database (NIST & GMD)	2	193	922	N.D.	N.D.	N.D.	N.D.	N.D.	N.D.	N.D.	N.D.	N.D.	N.D.	N.D.	N.D.	N.D.	N.D.	N.D.	N.D.	N.D.	N.D.	1.83E-02	3.02E-02	N.D.	N.D.	N.D.	N.D.	N.D.	N.D.	N.D.	N.D.
Xylulose (4TMS) isomer 2	25.6533	Standard	1	306		N.D.	1.48E-03	N.D.	N.D.	N.D.	4.71E-04	N.D.	N.D.	N.D.	N.D.	N.D.	N.D.	N.D.	N.D.	N.D.	N.D.	N.D.	N.D.	N.D.	5.18E-04	N.D.	N.D.	N.D.	N.D.	N.D.	N.D.	N.D.	N.D.
L-Asparagine (2TMS)	25.9858	Standard	1	159		N.D.	N.D.	N.D.	N.D.	N.D.	N.D.	N.D.	N.D.	1.97E-03	N.D.	N.D.	N.D.	N.D.	N.D.	N.D.	N.D.	N.D.	N.D.	6.29E-03	1.15E-03	N.D.	N.D.	N.D.	N.D.	1.48E-02	N.D.	N.D.	N.D.
2-Deoxypentitol (4TMS)	26.0009	Database (NIST)	3	219	823	N.D.	N.D.	N.D.	N.D.	N.D.	N.D.	N.D.	N.D.	6.34E-03	N.D.	N.D.	N.D.	N.D.	N.D.	N.D.	N.D.	N.D.	N.D.	N.D.	N.D.	N.D.	N.D.	N.D.	N.D.	N.D.	1.25E-02	N.D.	N.D.
Pyrogallol (3TMS)	26.0216	Database (NIST)	2	239	813	9.20E-03	3.91E-03	7.40E-03	1.67E-02	1.14E-02	8.92E-04	1.20E-02	1.89E-03	N.D.	1.46E-03	1.31E-02	4.77E-03	1.48E-03	2.34E-03	1.25E-02	3.98E-03	8.17E-03	1.87E-02	2.76E-03	1.69E-03	6.81E-03	2.84E-02	2.52E-02	4.11E-03	2.45E-03	6.13E-04	4.94E-03	6.79E-03
Ornithine (3TMS) isomer 1	26.4889	Standard	1	142		N.D.	N.D.	N.D.	N.D.	N.D.	N.D.	1.13E-01	N.D.	2.23E-03	N.D.	N.D.	N.D.	N.D.	N.D.	N.D.	N.D.	N.D.	N.D.	1.48E-03	1.09E-02	N.D.	N.D.	N.D.	N.D.	N.D.	N.D.	N.D.	N.D.
Phenylalanine (2TMS)	26.6665	Standard	1	218		1.03E-02	1.03E-01	3.20E-02	7.61E-03	2.38E-03	3.03E-02	3.60E-02	3.28E-03	2.34E-02	3.00E-03	1.22E-02	6.06E-03	1.97E-01	1.51E-01	1.17E-02	8.96E-02	2.02E-02	1.12E-01	2.80E-01	1.71E-01	3.37E-02	2.55E-02	6.78E-04	1.13E-02	9.30E-02	2.60E-02	3.47E-02	3.30E-02
Xylonic acid, 1,5-lactone (3TMS)	26.754	Database (NIST)	3	217	753	N.D.	N.D.	2.20E-04	N.D.	N.D.	N.D.	N.D.	N.D.	N.D.	N.D.	2.06E-03	5.16E-03	N.D.	1.12E-03	N.D.	5.01E-03	1.57E-04	1.24E-03	N.D.	N.D.	N.D.	N.D.	1.32E-03	N.D.	N.D.	N.D.	7.67E-04	N.D.
4-Hydroxybenzoic acid (2TMS)	26.8256	Database (NIST & Massbank)	2	267	864	2.05E-03	2.66E-03	2.79E-03	2.89E-03	1.94E-03	1.35E-03	4.03E-03	3.63E-03	3.39E-03	2.85E-03	2.14E-03	2.37E-03	3.76E-03	3.05E-03	3.39E-03	2.57E-03	3.35E-03	2.17E-03	1.70E-03	2.38E-03	2.10E-03	2.65E-03	1.63E-03	2.06E-03	1.87E-03	3.10E-03	2.56E-03	3.24E-03
Allantoin derivative 1	27.0981	Standard	1	403		N.D.	N.D.	6.87E-03	N.D.	N.D.	N.D.	1.62E-02	N.D.	N.D.	N.D.	N.D.	N.D.	8.52E-03	1.49E-01	N.D.	4.05E-02	1.61E-03	N.D.	9.92E-04	1.34E-02	1.62E-03	2.45E-02	N.D.	N.D.	4.35E-02	N.D.	N.D.	N.D.
Spermine per-TMS II	27.1002	Database (NIST)	3	160	680	1.10E-04	1.72E-02	N.D.	N.D.	N.D.	6.94E-03	N.D.	1.37E-02	1.19E-02	4.80E-03	9.18E-03	2.21E-03	N.D.	N.D.	2.52E-02	N.D.	2.09E-03	2.43E-02	N.D.	N.D.	8.23E-03	N.D.	N.D.	7.87E-03	N.D.	9.75E-03	2.70E-02	2.19E-02
Phloroglucinol (3TMS)	27.36	Database (NIST)	2	342	909	1.09E-01	2.36E-03	6.88E-03	2.64E-03	2.92E-03	6.93E-04	3.37E-03	4.87E-03	7.30E-04	9.08E-04	1.18E-03	4.07E-04	2.46E-04	N.D.	1.54E-04	1.72E-04	3.29E-04	7.00E-04	1.30E-03	1.44E-03	7.15E-04	4.42E-03	2.42E-03	1.51E-03	7.13E-04	8.23E-04	2.01E-03	1.01E-03
Gluconic acid (1MEOX 5TMS)	27.4495	Database (NIST)	3	204	848	6.63E-04	3.79E-02	4.98E-03	9.51E-03	3.41E-03	6.19E-04	7.74E-04	N.D.	1.45E-03	N.D.	7.06E-03	N.D.	2.38E-02	6.26E-04	N.D.	2.85E-03	2.25E-03	2.41E-02	9.59E-03	5.96E-03	4.70E-03	6.34E-03	5.13E-03	1.20E-03	2.81E-03	N.D.	1.48E-02	N.D.
3-Deoxy-pentonic acid (4TMS)	27.48	Database (NIST)	3	245	697	4.08E-03	4.54E-03	9.55E-03	7.04E-03	5.35E-03	1.60E-03	4.26E-03	5.00E-03	5.67E-03	N.D.	3.58E-03	1.49E-03	1.92E-02	1.19E-02	1.81E-02	1.06E-02	2.10E-02	2.63E-03	5.82E-03	4.86E-03	1.79E-02	1.48E-02	2.73E-03	6.77E-03	2.25E-02	6.32E-03	1.10E-02	N.D.
Lyxose (1MEOX 4TMS) isomer 1	27.5198	Standard	1	217		3.82E-02	3.51E-02	1.83E-01	4.21E-03	N.D.	2.44E-02	2.04E-02	N.D.	4.80E-03	2.66E-03	9.69E-04	7.81E-04	1.26E-01	1.58E-01	5.80E-04	1.42E-01	6.42E-03	2.70E-02	7.81E-02	1.06E-01	1.28E-02	9.02E-02	3.54E-02	N.D.	3.81E-02	2.62E-02	4.22E-02	5.02E-04
Lyxose (1MEOX 4TMS) isomer 2	27.927	Standard	1	217		6.46E-02	4.30E-03	N.D.	5.09E-02	1.39E-02	1.84E-02	3.54E-03	4.39E-03	1.41E-02	1.28E-03	N.D.	N.D.	1.10E-01	2.55E-02	6.54E-03	3.96E-02	1.11E-03	1.20E-02	N.D.	N.D.	8.02E-03	N.D.	N.D.	8.90E-03	2.31E-02	2.79E-02	N.D.	1.17E-02
Similar to Phloroglucinol (3TMS)	27.895	Database (NIST)	3	342	697	N.D.	9.61E-05	1.45E-03	N.D.	N.D.	1.42E-04	8.28E-05	N.D.	1.81E-03	N.D.	N.D.	N.D.	6.85E-04	5.25E-04	N.D.	2.35E-04	N.D.	4.91E-04	8.62E-05	9.17E-04	N.D.	1.61E-03	N.D.	N.D.	9.01E-03	N.D.	4.11E-04	1.65E-04
2,5-Furandicarboxylic acid (2TMS)	28.446	Database (NIST)	2	285	672	N.D.	N.D.	N.D.	1.61E-02	4.50E-03	N.D.	N.D.	N.D.	N.D.	N.D.	N.D.	N.D.	5.17E-03	N.D.	7.49E-03	N.D.	N.D.	N.D.	4.25E-04	4.44E-04	N.D.	N.D.	N.D.	5.87E-03	N.D.	N.D.	N.D.	N.D.
Arabinonic acid-1,4-lactone (3TMS)	28.4615	Database (NIST & GMD)	2	217	809	N.D.	N.D.	N.D.	N.D.	N.D.	N.D.	N.D.	8.78E-03	N.D.	N.D.	9.06E-05	N.D.	N.D.	N.D.	N.D.	N.D.	N.D.	N.D.	N.D.	N.D.	N.D.	N.D.	N.D.	N.D.	N.D.	N.D.	N.D.	N.D.
Hexestrol (2TMS)	28.5364	Database (NIST)	3	399	896	N.D.	N.D.	5.59E-05	N.D.	N.D.	N.D.	1.26E-03	N.D.	N.D.	N.D.	N.D.	N.D.	N.D.	9.08E-05	N.D.	5.94E-05	N.D.	N.D.	3.17E-04	5.85E-05	N.D.	N.D.	N.D.	N.D.	N.D.	N.D.	N.D.	N.D.
Levoglucosan (3TMS)	28.8666	Database (NIST & GMD)	2	204	898	2.86E-03	1.10E-03	2.70E-03	7.29E-03	6.72E-03	4.39E-04	3.65E-03	1.46E-02	2.95E-02	4.50E-03	2.10E-03	6.86E-04	1.18E-03	7.35E-04	1.28E-03	N.D.	8.10E-03	3.64E-03	N.D.	2.65E-04	5.46E-03	1.79E-03	7.36E-03	2.85E-03	5.20E-04	1.18E-03	1.56E-02	1.21E-02
Fucose (1MEOX 4TMS) isomer 1	29.2801	Database (NIST)	3	177	799	5.90E-02	2.30E-03	9.72E-03	4.37E-03	1.30E-02	6.03E-03	2.83E-02	3.29E-03	1.24E-02	4.01E-03	1.31E-02	4.64E-03	8.05E-03	2.16E-02	9.96E-03	1.31E-02	8.38E-03	1.63E-02	1.39E-02	9.88E-03	9.14E-03	1.27E-02	8.68E-03	7.46E-02	1.61E-02	8.46E-03	2.31E-02	7.50E-03
Xylitol (5TMS)	29.395	Standard	1	217		6.71E-02	4.37E-02	4.69E-02	5.56E-02	4.19E-02	2.79E-02	4.81E-02	2.95E-02	3.68E-02	3.04E-02	2.26E-02	1.18E-02	9.83E-02	5.38E-02	4.10E-02	2.97E-02	4.50E-02	1.41E-02	5.52E-02	4.19E-02	4.75E-02	5.79E-02	3.15E-02	3.17E-02	2.61E-02	3.90E-02	4.84E-02	1.62E-02
Ribitol (5TMS)	29.5139	Standard	1	217		1.96E-02	9.41E-03	2.09E-02	2.49E-02	1.28E-02	1.64E-02	2.77E-03	6.76E-03	2.36E-02	1.79E-03	1.66E-02	N.D.	2.22E-02	6.93E-03	2.89E-02	N.D.	1.04E-02	N.D.	N.D.	3.05E-04	1.30E-02	3.47E-02	5.46E-03	6.79E-03	1.07E-02	2.56E-02	4.03E-03	1.49E-02
Glycerol-2-phosphate (4TMS)	29.6075	Standard	1	299	811	N.D.	N.D.	N.D.	N.D.	N.D.	6.28E-04	N.D.	N.D.	3.57E-04	N.D.	1.36E-04	4.30E-04	N.D.	N.D.	N.D.	N.D.	N.D.	2.99E-03	N.D.	N.D.	N.D.	1.08E-03	N.D.	1.34E-03	1.02E-03	N.D.	N.D.	N.D.
Putrescine (4TMS)	29.6968	Standard	1	174		6.25E-03	3.61E-03	8.73E-03	2.36E-04	7.75E-03	1.67E-03	1.35E-02	2.83E-02	1.30E-03	3.78E-03	2.75E-03	1.40E-03	1.11E-01	N.D.	1.70E-02	N.D.	7.11E-03	1.11E-02	N.D.	N.D.	3.35E-03	2.50E-02	9.94E-03	3.44E-03	N.D.	2.13E-02	9.72E-03	2.12E-03
Ornithine (3TMS) isomer 2	30.164	Standard	1	186		N.D.	1.29E-03	4.83E-03	N.D.	N.D.	1.95E-04	1.93E-01	N.D.	2.20E-02	N.D.	N.D.	N.D.	8.51E-03	1.67E-03	N.D.	N.D.	N.D.	8.33E-03	2.67E-02	8.25E-02	N.D.	1.13E-03	N.D.	N.D.	7.35E-02	N.D.	1.16E-03	1.20E-03
cis-Aconitic acid (3TMS)	30.305	Standard	1	375		1.09E-04	6.57E-03	3.75E-03	N.D.	N.D.	N.D.	2.36E-02	5.37E-03	5.50E-03	N.D.	1.86E-03	9.80E-04	5.10E-04	1.85E-03	1.38E-02	2.53E-02	7.85E-03	N.D.	1.04E-03	1.01E-02	4.82E-03	N.D.	7.05E-04	6.52E-04	3.44E-02	N.D.	1.77E-02	2.84E-04
Vanillic acid (2TMS)	30.5492	Standard	1	297		N.D.	N.D.	N.D.	N.D.	N.D.	N.D.	N.D.	N.D.	N.D.	3.99E-03	N.D.	N.D.	N.D.	N.D.	N.D.	N.D.	N.D.	N.D.	N.D.	N.D.	N.D.	N.D.	N.D.	1.67E-03	N.D.	N.D.	N.D.	N.D.
Glycerol-3-Phosphate (4TMS)	30.5735	Standard	1	299		3.27E-04	N.D.	6.72E-03	4.50E-03	4.68E-03	2.26E-02	N.D.	6.17E-04	1.46E-02	3.89E-03	1.19E-02	1.20E-02	N.D.	N.D.	3.78E-03	N.D.	3.02E-04	6.17E-02	7.44E-03	5.76E-04	N.D.	1.70E-02	5.55E-03	3.10E-02	1.35E-02	1.57E-02	N.D.	6.40E-03
Allantoin derivative 2	30.6532	Standard	1	243		N.D.	N.D.	1.86E-02	N.D.	N.D.	N.D.	6.19E-02	N.D.	N.D.	N.D.	N.D.	N.D.	2.66E-02	2.66E-01	N.D.	9.78E-02	2.80E-02	N.D.	N.D.	3.33E-02	1.34E-02	5.67E-02	N.D.	N.D.	N.D.	N.D.	1.51E-02	N.D.
Methylfructoside (4TMS)	30.9187	Database (NIST)	3	257	790	2.66E-02	8.74E-04	1.16E-02	9.86E-02	1.41E-01	5.98E-03	1.10E-02	6.12E-02	2.02E-02	1.47E-02	4.63E-02	2.08E-02	N.D.	7.97E-03	7.79E-02	9.35E-03	2.95E-01	6.26E-02	N.D.	1.96E-04	1.83E-01	3.30E-02	1.84E-01	4.68E-02	9.24E-02	1.04E-02	9.73E-02	2.16E-02
σ-Coumaric acid (2TMS)	31.2649	Standard	1	293		2.20E-02	N.D.	N.D.	8.44E-02	1.86E-02	N.D.	4.84E-03	3.47E-03	N.D.	N.D.	N.D.	N.D.	2.22E-02	N.D.	3.28E-02	N.D.	5.42E-04	3.82E-03	5.35E-03	2.48E-03	N.D.	N.D.	N.D.	2.62E-02	5.15E-03	1.11E-03	2.23E-03	N.D.
β-D-Galactofuranose (5TMS)	31.6034	Database (NIST)	3	217	747	N.D.	N.D.	N.D.	N.D.	N.D.	N.D.	N.D.	N.D.	N.D.	N.D.	N.D.	N.D.	N.D.	N.D.	N.D.	N.D.	N.D.	N.D.	N.D.	N.D.	N.D.	N.D.	N.D.	N.D.	N.D.	N.D.	4.44E-02	N.D.
Fructose (5TMS) isomer 1	31.7068	Standard	1	437		3.59E-03	9.04E-02	3.13E-03	6.96E-04	N.D.	N.D.	1.52E-02	N.D.	3.23E-03	6.12E-04	1.91E-02	2.18E-03	9.77E-03	2.82E-02	8.43E-03	2.88E-02	4.84E-03	1.97E+00	2.14E-02	4.62E-02	3.36E-03	7.95E-03	3.45E-03	1.08E-03	4.24E-01	N.D.	1.03E-02	2.91E-04
Fructose (5TMS) isomer 2	31.8454	Standard	1	437		N.D.	5.28E-02	N.D.	N.D.	N.D.	N.D.	2.21E-03	N.D.	N.D.	5.23E-04	N.D.	N.D.	4.99E-04	N.D.	N.D.	5.64E-03	N.D.	1.53E+00	N.D.	N.D.	4.89E-04	N.D.	N.D.	1.22E-04	2.09E-01	N.D.	N.D.	N.D.
Ornithine (4TMS)	31.9012	Standard	1	142		N.D.	N.D.	1.08E-03	N.D.	N.D.	N.D.	1.71E-01	N.D.	N.D.	N.D.	N.D.	N.D.	3.01E-03	N.D.	N.D.	N.D.	N.D.	N.D.	5.49E-03	3.20E-02	N.D.	N.D.	N.D.	N.D.	N.D.	N.D.	N.D.	N.D.
Shikimic acid (4TMS)	32.0086	Standard	1	255		3.67E+00	2.78E-03	2.28E+00	2.39E+00	3.87E+00	1.84E-01	9.54E-01	1.34E+00	6.26E-02	1.87E-02	6.23E-01	2.21E+00	1.07E-02	5.29E-01	1.93E-01	1.16E+00	1.15E-03	1.99E+00	1.36E-03	N.D.	5.94E-02	3.70E+00	3.85E+00	7.68E-02	5.25E-03	1.72E-02	7.65E-03	1.03E-03
Protocatechuic acid (3TMS)	32.0424	Standard	1	370		N.D.	2.55E-03	N.D.	N.D.	N.D.	5.17E-04	N.D.	N.D.	9.84E-04	8.46E-04	1.37E-03	N.D.	3.37E-03	N.D.	9.40E-03	N.D.	2.79E-03	N.D.	2.11E-03	1.49E-03	1.63E-03	N.D.	N.D.	1.46E-03	5.76E-04	1.51E-03	2.51E-03	1.98E-03
Citric acid (4TMS)	32.1212	Standard	1	363		1.13E-01	9.18E-01	2.46E-01	1.56E-01	N.D.	2.83E-01	1.78E+00	5.86E-01	1.18E+00	5.21E-02	4.30E-01	1.23E-01	3.75E-01	6.84E-01	4.85E-01	1.70E+00	1.13E+00	7.40E-02	3.43E-01	5.21E-01	7.91E-01	N.D.	2.86E-02	4.42E-01	2.16E+00	1.45E-01	9.40E-01	6.32E-01
Isocitric acid (4TMS)	32.2169	Standard	1	245		1.24E-02	7.13E-03	1.75E-02	4.62E-02	1.26E-02	3.52E-04	N.D.	1.19E-02	5.87E-04	N.D.	3.50E-02	2.10E-02	2.17E-03	7.74E-02	1.36E+00	1.62E-02	1.38E-02	2.77E-02	7.72E-03	3.15E-02	2.53E-02	N.D.	1.30E-01	3.33E-02	N.D.	N.D.	6.71E-01	N.D.
Arginine [-NH3] (3TMS)	32.2295	Standard	1	256		N.D.	7.97E-04	N.D.	N.D.	N.D.	N.D.	6.35E-02	N.D.	N.D.	N.D.	N.D.	N.D.	1.20E-03	1.08E-03	N.D.	N.D.	N.D.	1.92E-03	4.06E-03	1.13E-02	N.D.	N.D.	N.D.	N.D.	N.D.	N.D.	N.D.	N.D.
Cholestan-3-one, dimethylhydrazone, (5α)	32.3853	Database (NIST)	3	428	793	N.D.	N.D.	5.09E-04	N.D.	N.D.	N.D.	4.90E-03	N.D.	N.D.	N.D.	N.D.	3.98E-05	8.96E-04	1.86E-02	1.48E-04	5.10E-03	6.27E-04	6.41E-04	1.11E-04	1.60E-03	6.04E-04	3.38E-03	N.D.	N.D.	N.D.	N.D.	6.46E-04	N.D.
2-Methylcitric acid (4TMS) isomer 1	32.5809	Standard	1	287		N.D.	N.D.	N.D.	N.D.	N.D.	N.D.	N.D.	N.D.	N.D.	N.D.	N.D.	2.12E-03	N.D.	N.D.	N.D.	N.D.	2.45E-03	N.D.	N.D.	N.D.	N.D.	N.D.	N.D.	N.D.	N.D.	N.D.	N.D.	N.D.
Galactaric acid (6TMS)	32.6431	Database (NIST)	3	292	566	N.D.	N.D.	N.D.	1.43E-03	3.17E-04	4.56E-03	1.33E-03	N.D.	8.80E-03	N.D.	N.D.	N.D.	N.D.	4.10E-03	N.D.	N.D.	N.D.	N.D.	N.D.	N.D.	N.D.	N.D.	N.D.	N.D.	N.D.	5.62E-03	N.D.	N.D.
Lysine (3TMS)	32.8334	Standard	1	200		7.13E-03	1.28E-01	3.85E-02	N.D.	5.46E-04	2.74E-02	5.68E-02	2.25E-04	2.04E-02	N.D.	N.D.	N.D.	2.79E-01	2.03E-01	8.55E-03	1.06E-01	1.31E-02	1.08E-01	4.01E-01	2.59E-01	2.73E-02	3.95E-02	N.D.	1.32E-03	1.18E-01	1.67E-02	3.22E-02	N.D.
Adenine (2TMS)	33.1523	Standard	1	264		1.15E-03	1.38E-02	4.72E-03	1.94E-04	N.D.	N.D.	3.06E-04	N.D.	N.D.	N.D.	N.D.	N.D.	2.27E-02	3.27E-02	N.D.	2.25E-02	4.43E-04	N.D.	2.26E-02	1.09E-02	1.34E-03	1.10E-03	1.65E-04	N.D.	1.95E-03	N.D.	N.D.	N.D.
Fructose (1MEOX 5TMS) isomer 1	33.4511	Standard	1	217		1.96E+00	3.83E+00	2.62E+00	3.29E+00	1.83E+00	7.99E-01	1.40E+00	4.43E-01	6.38E-01	9.25E-01	2.80E+00	4.52E-01	2.99E+00	1.52E+00	8.41E-01	1.43E+00	1.50E+00	3.24E+00	2.31E+00	2.31E+00	2.55E+00	3.44E+00	2.26E+00	1.01E+00	2.43E+00	1.77E+00	4.13E+00	7.73E-01
Estra-1,3,5(10)-trien-6-one, (16α,17β)- (3TMS)	33.4955	Database (NIST)	3	428	922	N.D.	N.D.	3.43E-04	N.D.	N.D.	N.D.	1.32E-03	N.D.	N.D.	N.D.	N.D.	N.D.	N.D.	2.39E-02	N.D.	8.81E-03	1.56E-03	N.D.	N.D.	7.02E-04	N.D.	5.18E-04	N.D.	N.D.	N.D.	N.D.	N.D.	N.D.
Fructose (1MEOX 5TMS) isomer 2	33.6752	Standard	1	217		1.44E+00	3.89E+00	2.13E+00	3.00E+00	1.37E+00	5.18E-01	1.05E+00	3.29E-01	4.98E-01	6.78E-01	2.32E+00	3.15E-01	2.97E+00	1.19E+00	6.19E-01	1.07E+00	1.11E+00	3.21E+00	1.81E+00	1.89E+00	1.92E+00	2.65E+00	1.79E+00	7.05E-01	1.98E+00	7.70E-01	3.80E+00	5.44E-01
Galactose (1MEOX 5TMS) isomer 1	33.8469	Standard	1	319		5.59E-01	1.57E-01	4.18E-01	6.98E-01	3.96E-01	1.07E-01	9.93E-02	2.96E-02	3.27E-01	3.83E-01	4.86E-02	6.85E-03	2.69E-01	5.70E-01	3.31E-01	4.97E-01	6.39E-02	2.97E-01	3.71E-01	1.94E-01	8.37E-02	1.06E+00	1.09E-01	3.13E-01	1.20E-01	1.38E-01	3.81E-01	1.11E-01
Glucose (1MEOX 5TMS) isomer 1	33.9195	Standard	1	319		1.70E+00	2.92E+00	2.29E+00	3.26E+00	1.67E+00	1.34E+00	1.31E+00	8.96E-01	1.46E+00	1.93E+00	1.27E+00	3.11E-01	3.09E+00	1.24E+00	1.64E+00	1.73E+00	1.90E+00	1.22E+00	2.31E+00	2.15E+00	1.61E+00	3.57E+00	1.76E+00	1.06E+00	2.02E+00	2.15E+00	4.44E+00	8.37E-01
Gluconic acid-1,4-lactone (4TMS)	34.1226	Standard	1	465		2.64E-03	N.D.	N.D.	3.67E-02	2.31E-02	N.D.	N.D.	N.D.	N.D.	N.D.	N.D.	N.D.	N.D.	N.D.	6.25E-02	N.D.	N.D.	N.D.	N.D.	N.D.	N.D.	N.D.	N.D.	3.10E-02	N.D.	N.D.	N.D.	N.D.
Galactose (1MEOX 5TMS) isomer 2	34.435	Standard	1	319		1.75E-01	1.02E-01	1.54E-01	5.52E-02	2.21E-03	N.D.	3.19E-02	3.68E-03	6.54E-03	N.D.	N.D.	2.05E-02	1.00E-01	2.49E-01	N.D.	2.31E-01	1.99E-01	N.D.	7.74E-02	1.49E-02	2.88E-03	6.48E-02	3.33E-02	3.88E-02	N.D.	N.D.	1.33E-01	N.D.
Glucose (1MEOX 5TMS) isomer 2	34.5246	Standard	1	319		9.03E-01	2.26E+00	1.70E+00	1.91E+00	7.22E-01	8.96E-01	5.18E-01	4.30E-01	2.98E-01	7.52E-01	9.17E-01	1.17E-01	2.03E+00	7.84E-01	5.47E-01	6.84E-01	4.75E-01	1.81E+00	1.27E+00	1.28E+00	1.08E+00	1.86E+00	1.12E+00	3.42E-01	1.05E+00	1.19E+00	2.45E+00	1.64E-01
L-Lysine (4TMS)	34.6474	Standard	1	317		5.37E-03	5.46E-02	1.50E-02	N.D.	4.14E-04	8.36E-04	2.00E-02	N.D.	N.D.	N.D.	N.D.	N.D.	1.37E-01	8.04E-02	N.D.	4.27E-02	1.65E-03	7.74E-02	1.56E-01	8.13E-02	4.95E-03	8.06E-03	N.D.	N.D.	N.D.	N.D.	N.D.	N.D.
Mannitol (6TMS) isomer 1	34.7737	Standard	1	319		8.50E-02	1.74E-02	1.17E-01	4.58E-02	6.73E-02	4.49E-02	2.17E-01	8.31E-02	6.92E-02	4.36E-02	2.92E-02	3.20E-02	7.24E-02	9.04E-02	1.40E-01	3.57E-02	6.55E-02	6.34E-02	1.14E+00	3.61E-01	4.92E-02	1.79E-01	4.02E-02	3.78E-02	4.90E-02	5.22E-02	6.28E-02	3.03E-02
2-Methylcitric acid (4TMS) isomer 2	34.7791	Database (NIST)	3	287	828	N.D.	N.D.	N.D.	N.D.	N.D.	N.D.	N.D.	N.D.	N.D.	N.D.	1.97E-03	1.04E-01	N.D.	N.D.	N.D.	N.D.	N.D.	1.31E-01	N.D.	N.D.	N.D.	N.D.	N.D.	N.D.	N.D.	N.D.	N.D.	N.D.
Similar to Gluconic acid-1,4-lactone (4TMS)	34.9368	Database (NIST)	3	465	779	4.23E-02	N.D.	N.D.	1.01E-01	N.D.	N.D.	N.D.	N.D.	N.D.	N.D.	N.D.	N.D.	4.19E-02	4.26E-03	N.D.	1.50E-02	N.D.	1.57E-03	1.43E-04	5.75E-04	N.D.	N.D.	N.D.	3.25E-02	N.D.	5.03E-04	N.D.	N.D.
L-Tyrosine (3TMS)	34.9989	Standard	1	218		N.D.	1.94E-02	8.36E-03	N.D.	N.D.	N.D.	6.72E-03	N.D.	N.D.	N.D.	N.D.	N.D.	1.19E-02	3.39E-02	N.D.	1.73E-02	1.03E-03	3.72E-02	4.85E-02	3.58E-02	N.D.	3.29E-03	N.D.	N.D.	N.D.	N.D.	N.D.	N.D.
p-Coumaric acid (2TMS)	35.0399	Standard	1	293		4.08E-02	N.D.	5.92E-03	1.94E-01	4.92E-02	2.21E-03	1.39E-02	9.85E-03	2.96E-03	N.D.	1.15E-03	2.57E-03	1.04E-02	N.D.	6.48E-02	N.D.	3.27E-03	5.74E-03	2.09E-02	1.17E-02	N.D.	6.99E-04	8.19E-04	N.D.	1.32E-02	3.35E-03	2.77E-03	4.13E-04
Mannitol (6TMS) isomer 2	35.1049	Standard	1	319		5.81E-04	N.D.	2.63E-03	N.D.	N.D.	N.D.	4.59E-04	N.D.	N.D.	N.D.	3.69E-04	7.48E-04	1.08E-03	N.D.	2.24E-03	N.D.	1.44E-03	N.D.	N.D.	N.D.	2.06E-03	N.D.	N.D.	N.D.	N.D.	N.D.	N.D.	N.D.
Hydrocaffeic acid (3TMS)	35.1515	Database (NIST & GMD)	2	398	897	1.34E-03	N.D.	8.04E-05	N.D.	N.D.	N.D.	1.92E-04	3.44E-04	N.D.	N.D.	2.17E-04	3.40E-04	4.74E-03	2.00E-04	1.27E-04	5.60E-04	5.88E-03	8.27E-04	5.57E-04	1.01E-03	4.53E-03	1.09E-03	3.79E-04	N.D.	N.D.	N.D.	N.D.	N.D.
L-Ascorbic acid (4TMS)	35.3558	Standard	1	332		2.84E-02	5.52E-01	1.92E-02	6.97E-02	2.27E-02	1.85E+00	7.20E-04	1.38E-02	2.33E+00	1.05E-04	N.D.	6.05E-03	1.84E-02	1.78E-03	3.25E-03	1.79E-03	2.26E-03	N.D.	1.84E-03	6.38E-04	2.96E-03	1.10E-02	4.43E-02	N.D.	1.22E+00	2.21E+00	1.97E-02	2.91E+00
Gallic acid (4TMS)	35.5052	Standard	1	458		4.40E-02	9.60E-05	1.90E-02	2.67E-03	2.50E-02	9.60E-05	1.46E-03	N.D.	N.D.	N.D.	1.52E-04	N.D.	N.D.	N.D.	N.D.	N.D.	N.D.	N.D.	7.89E-05	N.D.	2.19E-04	4.47E-02	2.08E-02	3.11E-04	N.D.	N.D.	N.D.	N.D.
Similar to Gluconic acid (6TMS) 1	35.6125	Database (NIST)	3	292	873	4.54E-03	4.63E-04	N.D.	1.05E-02	1.11E-03	N.D.	N.D.	5.95E-03	N.D.	N.D.	N.D.	N.D.	N.D.	4.10E-03	7.74E-04	2.91E-03	3.94E-03	N.D.	N.D.	1.98E-03	2.00E-03	N.D.	3.05E-03	N.D.	2.88E-03	N.D.	N.D.	N.D.
D-(+)-Arabitol (5TMS)	35.8448	Database (NIST)	3	394	790	8.66E-04	6.33E-03	2.38E-03	7.21E-03	3.30E-03	4.08E-03	1.74E-02	6.24E-03	1.00E-02	N.D.	5.44E-03	1.81E-03	1.26E-02	3.95E-03	1.25E-02	3.61E-03	2.16E-02	N.D.	3.99E-03	4.01E-03	3.23E-02	5.00E-03	7.11E-03	3.19E-03	5.63E-03	1.08E-02	2.94E-02	N.D.
Cis-caffeic acid (3TMS)	35.9679	Database (NIST & GMD)	2	368	925	1.50E-03	N.D.	5.35E-04	6.26E-03	2.92E-03	N.D.	1.11E-03	2.15E-02	N.D.	N.D.	4.05E-04	N.D.	1.10E-02	4.14E-04	3.30E-03	3.25E-04	7.67E-03	2.97E-04	2.51E-04	1.04E-04	6.69E-03	2.44E-04	6.23E-05	3.95E-03	N.D.	N.D.	2.43E-03	N.D.
Glucopyranose (5TMS)	36.2394	Database (NIST)	3	204	931	2.07E-02	1.65E-01	1.78E-02	2.28E-02	1.30E-02	2.04E-02	2.87E-02	6.65E-02	6.44E-02	2.54E-02	2.49E-02	7.06E-03	2.43E-02	9.39E-02	1.27E-01	9.45E-02	8.34E-02	2.28E+00	7.69E-02	1.58E-01	8.63E-02	1.98E-02	2.47E-02	3.55E-02	6.66E-01	1.69E-02	5.60E-02	2.93E-02
Similar to Gluconic acid (6TMS) 2	36.2744	Database (NIST)	3	292	907	9.96E-02	9.59E-02	7.95E-02	7.06E-02	2.16E-02	2.52E-02	8.81E-03	7.82E-02	1.30E-02	5.37E-04	6.39E-02	3.70E-02	4.25E-02	7.12E-02	2.19E-02	8.52E-02	1.46E-02	1.60E-01	3.23E-01	1.27E-02	2.32E-02	5.85E-03	3.95E-02	4.85E-02	2.35E-01	1.03E-03	5.42E-02	1.28E-02
Pantothenic acid (3TMS)	36.329	Database (NIST & GMD)	2	247	791	N.D.	N.D.	1.23E-03	N.D.	N.D.	N.D.	2.36E-04	N.D.	N.D.	4.25E-04	N.D.	N.D.	2.49E-03	2.36E-03	N.D.	N.D.	1.24E-03	N.D.	N.D.	3.22E-04	N.D.	N.D.	N.D.	N.D.	2.51E-03	N.D.	4.90E-04	N.D.
Similar to Gluconic acid (6TMS) 3	36.3542	Database (NIST)	3	292	883	8.41E-03	1.47E-02	9.85E-03	6.11E-02	9.52E-03	6.61E-03	2.80E-03	2.25E-03	2.91E-02	N.D.	4.76E-03	1.41E-03	4.02E-02	5.14E-03	1.29E-02	2.89E-04	8.24E-03	5.68E-03	N.D.	6.22E-03	4.37E-03	3.04E-02	1.84E-02	3.73E-03	2.28E-02	9.16E-03	1.25E-02	1.59E-02
Similar to Gluconic acid (6TMS) 4	36.4596	Database (NIST)	3	292	897	6.06E-03	N.D.	5.62E-03	9.94E-03	4.94E-03	6.70E-03	N.D.	N.D.	N.D.	N.D.	8.21E-03	N.D.	1.25E-02	3.88E-03	4.90E-03	N.D.	9.66E-03	6.13E-03	N.D.	5.31E-03	5.83E-03	1.47E-02	N.D.	3.80E-03	9.34E-03	1.75E-03	N.D.	1.16E-03
4-Methylcinnamic acid (2TMS)	36.6129	Database (NIST)	3	338	728	N.D.	N.D.	N.D.	N.D.	N.D.	N.D.	N.D.	N.D.	N.D.	N.D.	N.D.	N.D.	N.D.	N.D.	N.D.	N.D.	2.31E-03	N.D.	N.D.	N.D.	1.79E-03	N.D.	N.D.	N.D.	N.D.	N.D.	N.D.	N.D.
Gluconic acid (6TMS)	36.8781	Standard	1	292		1.45E-02	6.52E-04	1.75E-02	N.D.	7.17E-03	1.47E-02	1.30E-02	1.00E-02	7.72E-03	N.D.	2.91E-03	7.14E-03	9.86E-03	1.97E-02	9.23E-03	1.25E-02	6.50E-03	8.92E-03	2.04E-02	2.35E-02	1.21E-02	4.49E-02	8.74E-03	N.D.	2.38E-02	8.64E-03	1.89E-02	6.08E-03
Inositol, scyllo (6TMS)	37.0687	Database (NIST & GMD)	2	318	902	2.64E-03	1.08E-01	6.56E-02	N.D.	N.D.	5.32E-04	1.15E+00	3.39E+00	N.D.	2.14E-03	1.85E+00	2.35E+00	2.62E+00	1.99E+00	3.23E+00	2.26E+00	3.31E+00	2.29E+00	7.37E-01	1.01E+00	3.22E+00	1.16E-01	1.18E-04	2.76E+00	4.73E-01	2.01E-03	3.02E+00	2.57E-03
Catechollactate (4TMS)	37.9558	Database (NIST)	3	267	918	N.D.	N.D.	1.15E-03	1.75E-03	2.08E-03	N.D.	4.44E-05	9.71E-04	N.D.	N.D.	2.82E-04	1.19E-03	3.21E-01	6.10E-04	8.65E-05	1.32E-03	3.26E-01	2.20E-03	6.00E-03	2.93E-03	3.22E-01	2.60E-03	1.29E-03	5.15E-05	1.15E-04	N.D.	N.D.	3.59E-04
Dopamine (3TMS)	38.2556	Standard	1	174		6.00E-04	2.25E-04	8.86E-04	N.D.	8.25E-04	1.28E-04	3.56E-04	7.50E-05	7.24E-05	1.01E+00	1.05E-01	N.D.	7.53E-02	N.D.	N.D.	N.D.	3.02E-02	1.18E-03	7.82E-05	N.D.	4.86E-02	1.32E-03	5.20E-04	1.65E-01	2.79E-02	N.D.	1.24E-03	1.44E-04
Ferulic acid (2TMS)	38.666	Standard	1	338		8.58E-03	2.08E-04	2.07E-04	3.31E-02	6.60E-03	3.45E-04	5.05E-03	5.07E-03	1.25E-04	N.D.	4.90E-04	2.01E-02	6.32E-03	2.23E-03	2.70E-04	3.20E-03	1.67E-02	3.45E-03	1.02E-02	2.95E-03	8.00E-03	N.D.	N.D.	1.29E-03	1.48E-03	2.80E-03	2.66E-04	5.59E-04
Sedoheptulose (1MEOX 6TMS)	38.9789	Database (NIST)	3	319	819	1.21E-02	1.01E-01	2.70E-02	2.63E-02	1.64E-02	3.02E-02	5.25E-02	1.33E-02	3.40E-02	2.11E-02	1.15E-02	1.99E-02	3.45E-02	2.90E-02	2.55E-02	3.82E-02	1.48E-02	1.76E-02	9.07E-02	8.62E-03	6.34E-02	1.39E-02	3.23E-02	1.27E-02	6.42E-03	6.81E-02	1.88E-02	2.37E-02
Norepinephrine (5TMS)	40.0127	Database (NIST & HMDB)	2	355	909	N.D.	N.D.	N.D.	N.D.	N.D.	N.D.	N.D.	N.D.	N.D.	1.59E-01	N.D.	N.D.	N.D.	N.D.	N.D.	N.D.	N.D.	N.D.	N.D.	N.D.	N.D.	N.D.	N.D.	1.40E-03	N.D.	N.D.	N.D.	N.D.
Indolelactic acid (3TMS)	40.3231	Database (NIST & HMDB)	2	202	882	N.D.	N.D.	N.D.	N.D.	N.D.	N.D.	N.D.	3.46E-03	N.D.	N.D.	N.D.	N.D.	N.D.	N.D.	N.D.	N.D.	N.D.	N.D.	N.D.	N.D.	N.D.	N.D.	N.D.	N.D.	N.D.	N.D.	N.D.	N.D.
Tryptophan (2TMS)	40.9577	Standard	1	202		4.15E-04	1.65E-02	4.14E-03	N.D.	N.D.	5.21E-04	2.07E-02	N.D.	N.D.	N.D.	1.28E-04	N.D.	3.09E-02	1.26E-02	N.D.	3.75E-03	6.76E-04	1.41E-02	5.00E-02	2.90E-02	4.40E-04	2.84E-03	N.D.	N.D.	3.43E-03	N.D.	3.35E-03	N.D.
Sinapic acid (2TMS)	42.055	Standard	1	368		N.D.	N.D.	4.04E-04	N.D.	N.D.	N.D.	N.D.	5.93E-04	1.70E-03	9.67E-05	N.D.	N.D.	6.62E-03	N.D.	N.D.	2.50E-03	6.79E-03	4.41E-04	1.78E-04	N.D.	3.08E-04	4.28E-04	N.D.	N.D.	8.96E-05	2.82E-03	2.15E-04	4.20E-04
Inositol-2-phopsphate , myo- (7TMS)	45.4494	Database (NIST & GMD)	2	318	879	1.05E-03	2.54E-02	4.30E-03	7.74E-04	4.41E-04	5.26E-03	5.51E-03	1.13E-03	3.63E-03	1.76E-03	6.40E-03	1.23E-02	7.72E-04	6.17E-03	1.07E-02	6.46E-03	7.40E-03	2.19E-02	8.49E-03	2.88E-03	1.12E-02	1.97E-03	1.00E-03	2.88E-03	4.16E-02	8.74E-03	1.02E-03	1.23E-03
Uridine (3TMS)	46.1596	Standard	1	259		N.D.	1.60E-03	1.85E-03	N.D.	N.D.	N.D.	N.D.	N.D.	N.D.	N.D.	N.D.	N.D.	3.06E-03	2.94E-02	N.D.	1.24E-02	N.D.	4.37E-04	1.86E-02	3.73E-03	N.D.	N.D.	N.D.	N.D.	4.36E-03	N.D.	N.D.	N.D.
Arbutin (5TMS)	48.9615	Database (NIST & GMD)	2	254	819	N.D.	N.D.	N.D.	1.61E-04	1.12E-04	2.40E-04	N.D.	1.64E-04	7.47E-04	4.82E-03	N.D.	2.74E-03	N.D.	N.D.	N.D.	N.D.	N.D.	3.10E-04	4.13E-03	3.28E-03	N.D.	N.D.	N.D.	N.D.	N.D.	4.34E-04	7.04E-05	3.58E-04
Similar to Sucrose (8TMS) 1	48.9762	Database (NIST)	3	437	717	N.D.	N.D.	N.D.	2.63E-03	2.05E-03	5.31E-04	9.01E-04	1.44E-03	1.09E-02	N.D.	1.89E-04	9.14E-04	N.D.	N.D.	2.90E-03	N.D.	1.48E-04	4.37E-04	N.D.	N.D.	N.D.	2.03E-03	2.17E-04	4.57E-03	5.24E-03	6.73E-03	N.D.	7.92E-03
1-Monopalmitin (2TMS)	49.0701	Database (NIST)	3	371	860	1.30E-03	9.13E-04	2.25E-03	1.22E-03	1.56E-03	1.15E-03	8.45E-04	N.D.	1.59E-03	2.02E-03	1.01E-03	2.90E-03	1.22E-03	1.78E-03	1.15E-03	4.27E-03	1.79E-03	1.87E-03	1.00E-03	2.29E-03	2.49E-03	N.D.	4.13E-03	1.42E-03	7.63E-03	6.97E-03	7.37E-03	4.95E-03
Sucrose (6TMS)	49.8453	Standard	1	361		1.05E+00	1.78E+00	1.06E+00	2.10E+00	2.91E+00	1.50E+00	1.94E+00	3.41E+00	2.55E+00	2.52E+00	3.14E+00	3.26E+00	8.06E-01	7.57E-01	2.83E+00	7.51E-01	2.28E+00	2.04E+00	1.87E-01	3.07E-01	2.92E+00	2.19E+00	1.90E+00	3.03E+00	1.72E+00	2.65E+00	2.60E+00	4.32E+00
Similar to Shikimic acid (4TMS)	50.1306	Database (NIST)	3	462	742	2.90E-03	N.D.	1.18E-02	N.D.	N.D.	N.D.	N.D.	N.D.	N.D.	N.D.	N.D.	N.D.	N.D.	N.D.	N.D.	N.D.	N.D.	4.93E-03	N.D.	N.D.	N.D.	N.D.	N.D.	N.D.	N.D.	N.D.	N.D.	N.D.
Monostearin (2TMS)	52.615	Database (NIST)	3	399	854	2.45E-04	4.07E-04	9.01E-04	5.63E-05	3.80E-04	N.D.	3.13E-04	N.D.	2.17E-04	9.54E-04	2.68E-04	3.32E-03	1.67E-04	5.71E-04	1.00E-04	1.09E-03	6.98E-04	N.D.	3.49E-04	7.74E-04	3.65E-04	8.70E-04	6.25E-04	6.86E-05	1.26E-03	1.43E-03	1.83E-03	9.53E-04
Epicatechin (5TMS)	53.8408	Standard	1	368		N.D.	N.D.	N.D.	4.84E-04	1.11E-04	N.D.	2.23E-01	1.46E-01	2.43E-04	N.D.	1.02E-01	1.80E+00	2.39E-02	9.98E-03	N.D.	7.65E-04	N.D.	N.D.	N.D.	1.35E-04	N.D.	1.01E-04	N.D.	3.68E-02	N.D.	N.D.	5.75E-01	N.D.
Maltose (1MEOX 8TMS)	53.9376	Database (NIST & GMD)	2	361	791	1.22E-02	4.06E-04	5.30E-02	6.93E-03	N.D.	N.D.	1.21E-03	N.D.	N.D.	2.92E-04	N.D.	N.D.	3.25E-02	1.46E-02	N.D.	3.26E-03	N.D.	1.14E-02	N.D.	N.D.	N.D.	6.65E-02	1.62E-02	N.D.	N.D.	1.39E-03	2.83E-02	N.D.
Catechin (5TMS)	54.2591	Standard	1	368		4.15E-03	N.D.	N.D.	N.D.	N.D.	1.43E-04	2.32E+00	1.78E+00	2.31E-04	N.D.	7.22E-04	4.27E-01	3.88E-03	N.D.	N.D.	N.D.	N.D.	1.63E-03	7.18E-03	2.56E-02	N.D.	4.47E-04	N.D.	N.D.	4.07E-04	N.D.	7.94E-03	N.D.
Gallocatechin (5TMS)	54.9928	Standard	1	456		3.39E-02	N.D.	1.49E-03	4.76E-02	6.93E-03	N.D.	3.08E-01	2.16E-01	N.D.	N.D.	N.D.	7.11E-05	N.D.	N.D.	N.D.	N.D.	N.D.	N.D.	N.D.	N.D.	N.D.	1.56E-03	6.95E-04	N.D.	N.D.	N.D.	1.94E-01	N.D.
Melibiose (8TMS) isomer 1	58.3839	Database (NIST)	3	361	826	4.74E-02	7.16E-02	1.24E-01	1.63E-02	9.36E-03	2.65E-01	4.17E-02	8.54E-03	2.01E-01	2.40E-02	5.20E-02	1.75E-02	2.39E-02	9.24E-02	1.16E-02	4.29E-01	6.26E-02	6.23E-01	1.09E-01	1.64E-02	6.17E-02	2.48E-02	3.39E-02	4.60E-01	8.49E-03	2.76E-01	3.09E-01	2.95E-01
Similar to Caffeic acid (3TMS) 1	58.8334	Database (NIST)	3	396	785	N.D.	N.D.	N.D.	N.D.	N.D.	N.D.	N.D.	N.D.	N.D.	N.D.	N.D.	N.D.	2.93E-03	N.D.	N.D.	N.D.	N.D.	N.D.	N.D.	N.D.	N.D.	N.D.	N.D.	N.D.	N.D.	N.D.	N.D.	N.D.
Quercetin (5TMS)	59.0218	Database (NIST & GMD)	2	575	808	N.D.	N.D.	3.17E-03	7.98E-05	8.75E-05	N.D.	2.49E-04	N.D.	N.D.	N.D.	N.D.	1.74E-02	N.D.	N.D.	N.D.	N.D.	N.D.	N.D.	N.D.	N.D.	N.D.	N.D.	2.03E-03	N.D.	N.D.	N.D.	3.74E-03	N.D.
Similar to Caffeic acid (3TMS) 2	59.9191	Database (NIST)	3	396	712	N.D.	N.D.	N.D.	N.D.	N.D.	N.D.	N.D.	N.D.	N.D.	N.D.	N.D.	N.D.	1.07E-03	N.D.	N.D.	N.D.	1.15E-03	N.D.	N.D.	N.D.	1.43E-03	N.D.	N.D.	N.D.	N.D.	N.D.	N.D.	N.D.
Similar to Sucrose (8TMS) 2	62.2223	Database (NIST)	3	361	871	1.06E-03	6.26E-04	4.24E-03	1.70E-03	3.28E-03	8.86E-04	3.98E-03	1.20E-04	1.40E-03	N.D.	6.91E-04	8.18E-02	1.75E-03	N.D.	1.87E-04	9.60E-04	N.D.	2.34E-04	N.D.	N.D.	1.66E-03	9.66E-03	1.01E-02	4.16E-04	N.D.	9.86E-04	7.78E-03	N.D.
Similar to Caffeic acid (3TMS) 3	62.7086	Database (NIST)	3	396	760	N.D.	N.D.	N.D.	N.D.	N.D.	N.D.	N.D.	N.D.	N.D.	N.D.	N.D.	N.D.	4.64E-03	N.D.	N.D.	N.D.	2.01E-02	N.D.	N.D.	N.D.	1.99E-02	N.D.	N.D.	N.D.	N.D.	N.D.	N.D.	N.D.

*Abundance expressed relative to internal standard

** Matches to NIST database showed a probability ≥ 80%

N.D. - Metabolite not detected

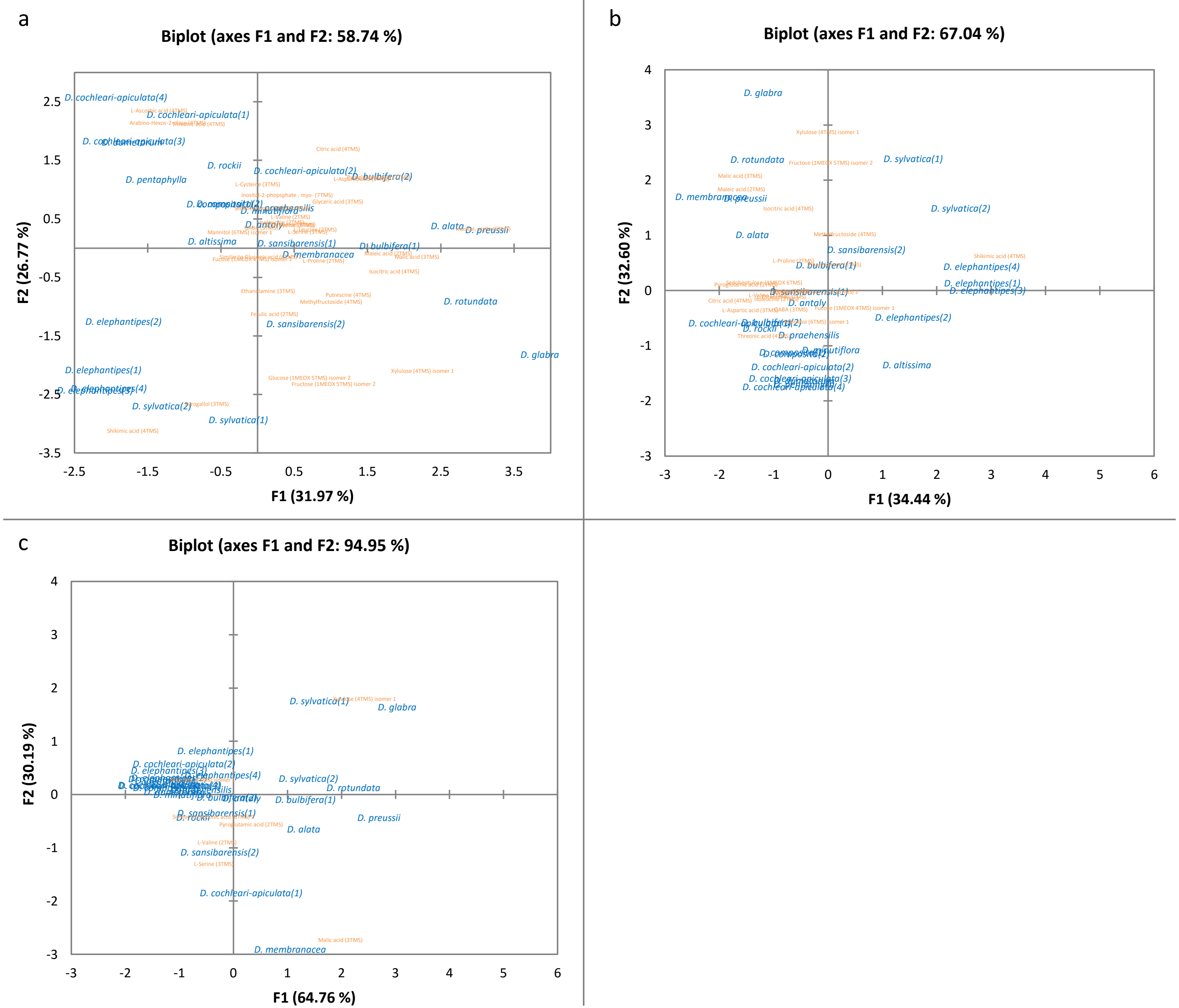

Supplementary Figure S5. Consensus GPA bi-plots (n=6) on the polar fraction of leaf extracts from the 28 *Dioscorea* accessions using metabolites which following Bonferroni-corrected Conover-Iman post hoc ($p<0.0001$) following two-tailed Kruskal-Wallis' one-way analysis of variance discriminate (a) ≥ 11 groups, (b) ≥ 13 groups and (c) ≥ 15 groups.

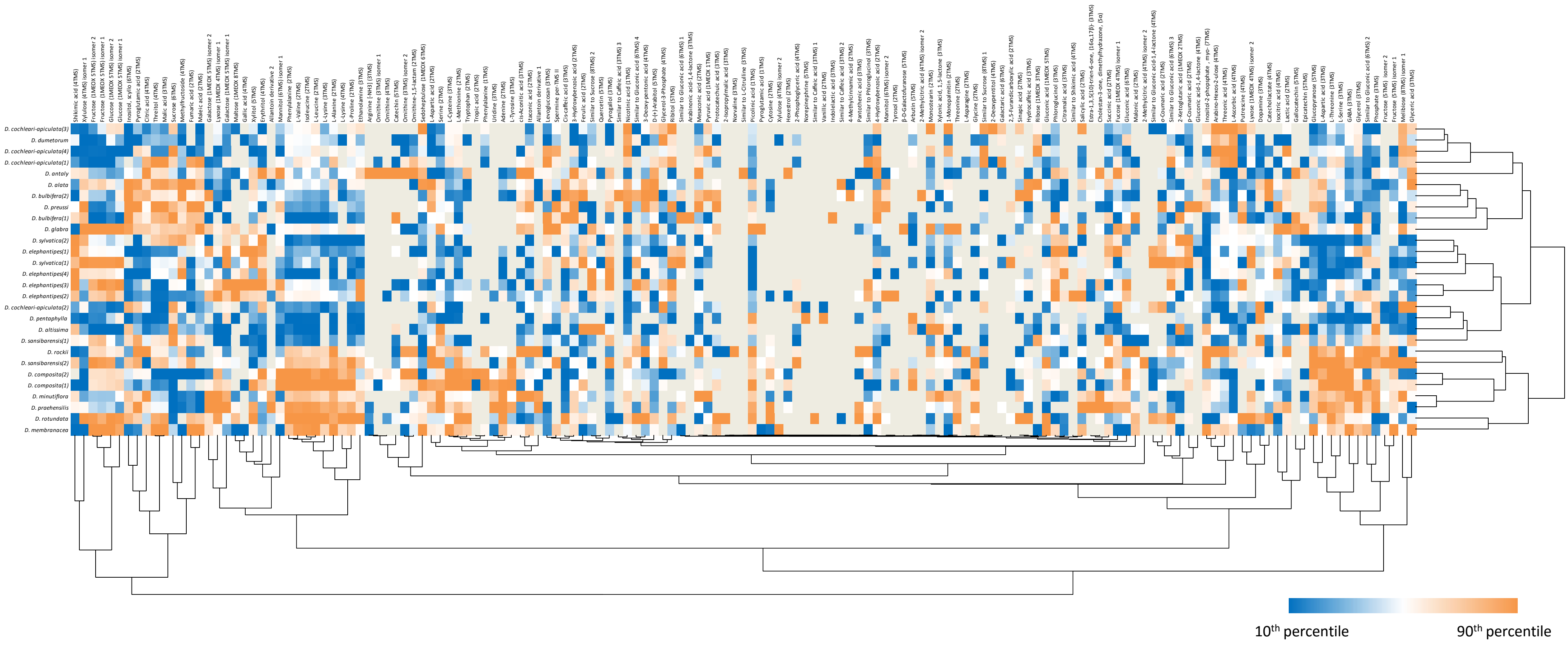

Supplementary Figure S6. Dendrogram generated from the complete-linkage clustering of mean (n=6) metabolite profiles for the *Dioscorea* samples analysed, showing the clustering of biochemically-related compounds. The heat-map shows qualitative and quantitative differences in metabolic compositions between species. Each row represents an accession, as per Figure 2. Each column represents a metabolite. Cells are coloured based on normalised (range-scaled) metabolite abundances. Grey squares indicate when a metabolite was not detected. The heat-map has been produced in Microsoft Excel.

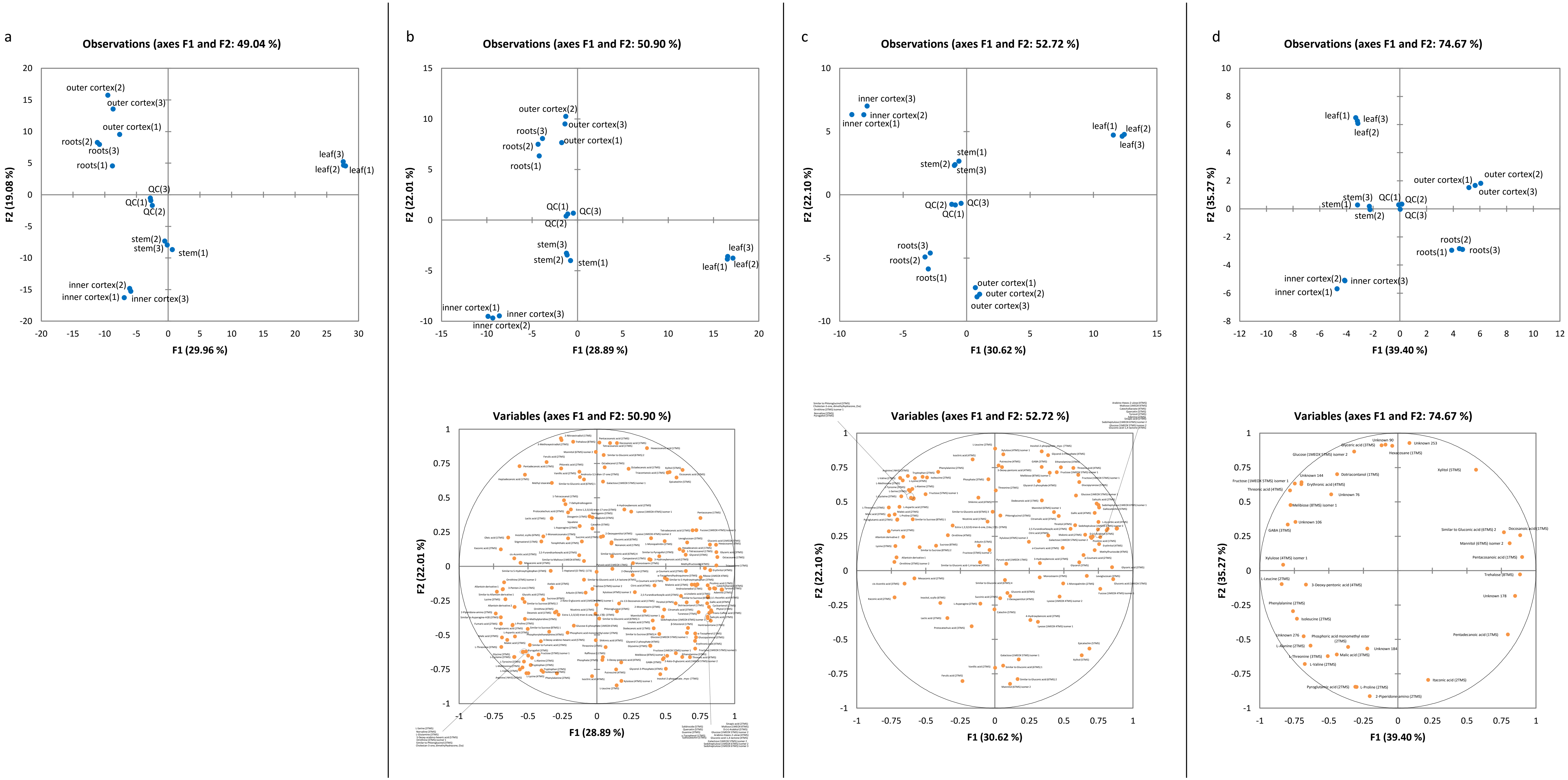

Supplementary Figure S7. PCA plots (above: variables plot; below: loadings plot) following metabolite profiling on different sections of *D. elephantipes* show similar differentiation using (a) all features; (b) known and putative features; (c) known features and (d) metabolites which discriminated all 5 sections after Bonferroni-corrected Conover-Iman post hoc ($p<0.0001$) following two-tailed Kruskal-Wallis' one way analysis of variance.

Supplementary Methods. Further explanation of statistical analyses.

Generalised Procrustes Analysis (GPA) is a multi-block method to analyse multiple tables of variables recorded on the same set of objects. The method achieves a consensus configuration of objects by applying Procrustes transformation to each variables table and then Principal Component Analysis (PCA) on the covariance matrix of the consensus (mean average) of the transformed data tables. GPA was applied in this study to generate a consensus configuration as pooled quality control (QC) samples were not present due to limited amounts of material. The consensus was validated via permutation testing (300 simulations). GPA was favoured over Multiple Factor Analysis (MFA) as GPA retains the relative distances of objects in each individual variables table. Procrustes transformation has the addition benefit of making each individual variable table as alike as possible and thus negates some differences in measurements that arise from the analytical platform itself.

Univariate normality testing (Shapiro-Wilk) showed a non-normal distribution for all metabolites; as is common in metabolomics data sets. Additionally, due to the small sample size (6 replicates), it was chosen that further analysis was conducted using robust non-parametric tests and the conservative Bonferroni correction to control the familywise error rate.

Agglomerative Hierarchical Clustering (AHC) via Spearman dissimilarity with complete linkage was performed on mean-averaged, centred data for all consistently-measured metabolites (Figure 2, Supplementary Fig. S6).
